# Statistical Analysis of Readthrough Levels for Nonsense Mutations in Mammalian Cells Reveals a Major Determinant of Response to Gentamicin

**DOI:** 10.1371/journal.pgen.1002608

**Published:** 2012-03-29

**Authors:** Célia Floquet, Isabelle Hatin, Jean-Pierre Rousset, Laure Bidou

**Affiliations:** 1 Université Paris-Sud, Institut de Génétique et Microbiologie, UMR8621, Orsay, France; 2 CNRS, Orsay, France; 3 UPMC Université Paris VI, Paris, France; University of Utah Hospital School of Medicine, United States of America

## Abstract

The efficiency of translation termination depends on the nature of the stop codon and the surrounding nucleotides. Some molecules, such as aminoglycoside antibiotics (gentamicin), decrease termination efficiency and are currently being evaluated for diseases caused by premature termination codons. However, the readthrough response to treatment is highly variable and little is known about the rules governing readthrough level and response to aminoglycosides. In this study, we carried out in-depth statistical analysis on a very large set of nonsense mutations to decipher the elements of nucleotide context responsible for modulating readthrough levels and gentamicin response. We quantified readthrough for 66 sequences containing a stop codon, in the presence and absence of gentamicin, in cultured mammalian cells. We demonstrated that the efficiency of readthrough after treatment is determined by the complex interplay between the stop codon and a larger sequence context. There was a strong positive correlation between basal and induced readthrough levels, and a weak negative correlation between basal readthrough level and gentamicin response (i.e. the factor of increase from basal to induced readthrough levels). The identity of the stop codon did not affect the response to gentamicin treatment. In agreement with a previous report, we confirm that the presence of a cytosine in +4 position promotes higher basal and gentamicin-induced readthrough than other nucleotides. We highlight for the first time that the presence of a uracil residue immediately upstream from the stop codon is a major determinant of the response to gentamicin. Moreover, this effect was mediated by the nucleotide itself, rather than by the amino-acid or tRNA corresponding to the −1 codon. Finally, we point out that a uracil at this position associated with a cytosine at +4 results in an optimal gentamicin-induced readthrough, which is the therapeutically relevant variable.

## Author Summary

Nonsense mutations are single-nucleotide variations within the coding sequence of a gene that result in a premature termination codon. The presence of such mutations leads to the synthesis of a truncated protein unable to fulfill its normal function. Over the last ten years, treatment strategies have emerged based on the use of molecules, such as aminoglycoside antibiotics (gentamicin) that facilitate the readthrough of premature termination codons, thus restoring the synthesis of a full-length protein. Such strategies have been tested for various genetic diseases, including Duchenne muscular dystrophy and cystic fibrosis. The readthrough level depends on the nature of the stop codon and the surrounding nucleotide context, but little was known of the rules governing readthrough level and response to aminoglycosides. In this study, we use a large set of nonsense mutations for an in-depth statistical analysis designed to decipher the element of the nucleotide context responsible for modulating readthrough levels. We analyse the impact of the six nucleotides upstream and downstream from the stop codon. We demonstrate that the presence of a uracil residue immediately upstream the stop codon is associated with a stronger response to gentamicin treatment than the presence of any of the other three nucleotides.

## Introduction

Translation is terminated by a stop codon entering the A site of the ribosome, inducing the release of the polypeptide chain from the peptidyl-t-RNA [1]. Two polypeptide chain release factors have been identified in eukaryotes: eRF1 (eukaryotic Release Factor one), which recognizes all three nonsense codons, and eRF3 (eukaryotic Release Factor three) which stimulates polypeptide release from the ribosome in a GTP and eRF1-dependent manner [2]. Under normal conditions, translation termination at natural termination codons is a very efficient process, with an estimated error rate of 0.01 to 0.1% in mammalian cells (unpublished data). However, near-cognate aminoacyl-tRNAs (with pairing of two of the three bases) can compete with eRF1 for stop codon binding, resulting in translational readthrough. The rules governing readthrough efficiency are far from clear, but readthrough levels have been shown to depend on the type of stop codon, with UAA being a better terminator than UAG and UGA, and even more strongly on the surrounding nucleotide context [3]–[6]. The effect of nucleotide context on readthrough level has been extensively studied in yeast: Bonetti and coworkers demonstrated that upstream and downstream components act in synergy to determine readthrough efficiency [7]. Two studies based on the screening of a degenerate oligonucleotide library established a consensus sequence, *NAA* ***STOP*** *CA (A/G) N (U/C/G) A*, promoting high levels of readthrough [6], [8]. Less is currently known about the effect of sequence context on readthrough levels in mammalians cells, because only a small number of contexts surrounding stop codons have been investigated.

Readthrough can be stimulated by aminoglycoside antibiotics, such as gentamicin, making it possible to generate a full-length protein from genes carrying a nonsense mutation [9], [10]. These antibiotics interact with the highly conserved decoding center of ribosomal RNA, promoting the recognition of the stop codon by a near-cognate tRNA [11], [12]. A number of studies and clinical trials have investigated the possible use of this antibiotic for the treatment of human diseases resulting from the presence of a premature termination codon (PTC) in a particular gene (for review, see [13], [14]). However, basal and induced readthrough efficiencies differ considerably between nonsense mutations [3], [15] so only a subset of patients would be likely to benefit from gentamicin treatment. Moreover, due to the complexity of the mechanisms involved, it is not possible to predict readthrough efficiency from the nucleotide context of the nonsense mutation. It is crucial to determine the patients most likely to benefit from treatment, and it is currently necessary to measure the readthrough level of each nonsense mutation in cell culture, as readthrough levels in culture are correlated with those *in vivo* [16]. Many studies have indicated that the nucleotide immediately downstream from the stop codon (defined as +4) is a crucial determinant of termination efficiency in eukaryotes [17]. Moreover, this nucleotide has been shown to crosslink with release factor class I [18]. A cytosine (C) residue in the +4 position generally promotes higher levels of readthrough in the presence or absence of aminoglycosides. However, some nonsense mutations with a C residue in the +4 position may display moderate levels of readthrough [3]. Thus, the identity of the nucleotide immediately downstream from the stop codon is not sufficient to predict readthrough efficiency for a given nonsense mutation. No systematic study has been performed and our knowledge of the effects of nucleotide context on readthrough level and gentamicin response (i.e. the factor of increase between basal readthrough and drug-induced readthrough) is therefore incomplete.

We used a set of 66 sequences, each containing a stop codon - mostly nonsense mutations implicated in various human diseases - inserted into the same reporter vector for an extensive statistical analysis of the determinants of readthrough levels and gentamicin response. We found a strong correlation between basal readthrough level and antibiotic-induced readthrough level and a very weak negative correlation between basal readthrough level and gentamicin response. The nature of the stop codon did not affect the sensitivity of the nonsense mutation to gentamicin treatment. A comprehensive analysis of the surrounding nucleotides identified positions playing an important role in determining readthrough levels and gentamicin response. In particular, we demonstrated that the nucleotide immediately upstream from the stop codon was a major determinant of gentamicin response and that this effect was mediated by the nucleotide itself, rather than by the nature of the last amino acid or the tRNA present in the ribosomal P-site.

Based on these findings, we have developed the first rules for predicting the sensitivity of nonsense mutations to aminoglycoside treatments based on the surrounding nucleotide sequence.

## Results/Discussion

### Readthrough quantification for 66 sequences containing a stop codon

**Figure 1:**
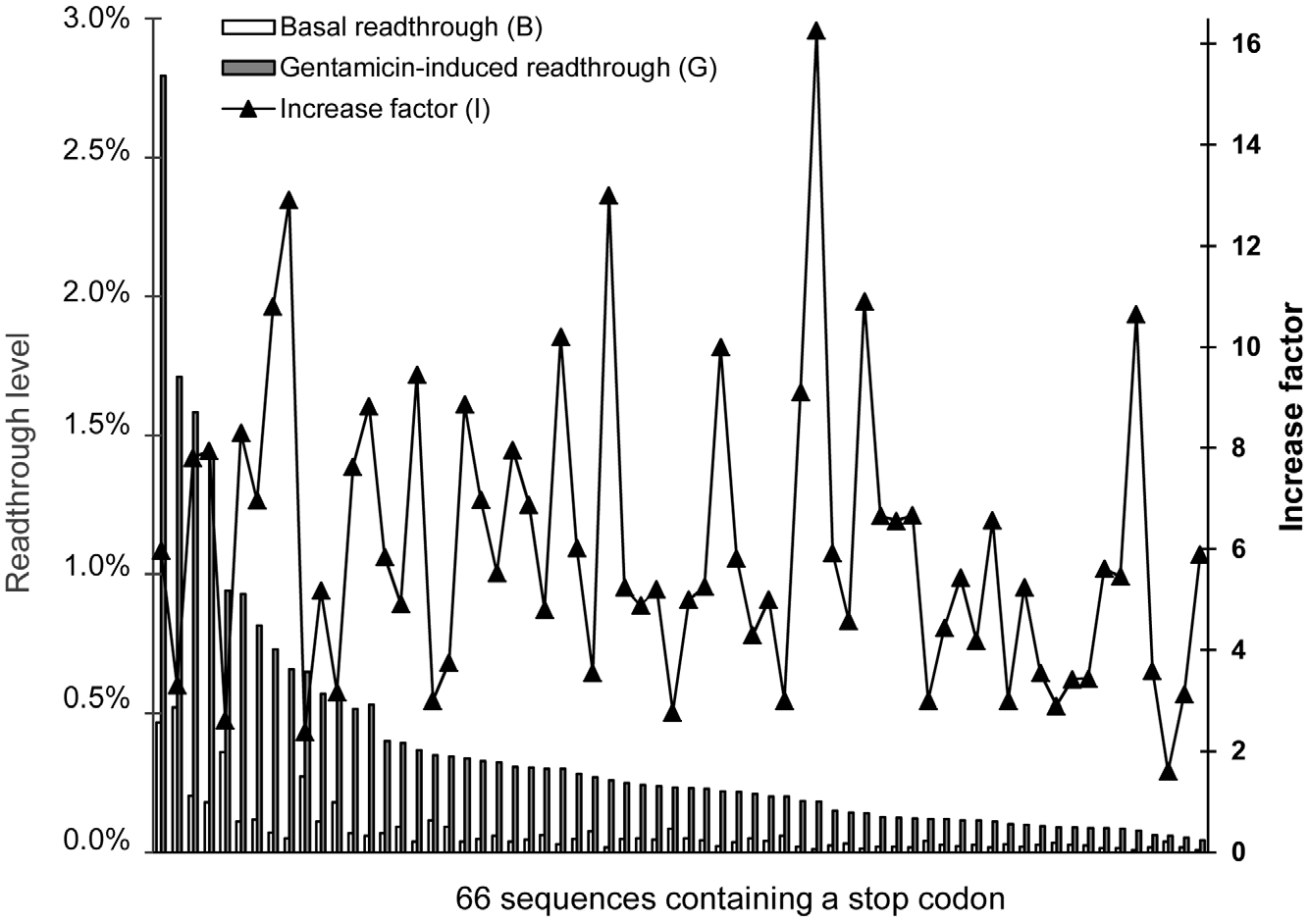
Readthrough levels and factor of increase for 66 sequences containing a stop codon. Readthrough efficiencies of 66 sequences (65 nonsense mutations and 1 natural stop codon) were measured in NIH3T3 cells with and without gentamicin (800 µg/ml) treatment for 24 H. Each value shown is the mean of at least three independent experiments. For each sequence, the factor of increase (I) is the ratio of the gentamicin-induced readthrough level (G) to the basal readthrough level (B). Sequences are ranked in descending order of gentamicin-induced readthrough level.

We analyzed readthrough levels for 66 stop codons, including one natural termination codon and 65 nonsense mutations implicated in various diseases (Figure 1 and Table S1): The CFTR gene for cystic fibrosis [19], the dystrophin gene for Duchenne muscular dystrophy [3], the LAMA-2 gene for congenital muscular dystrophy [16], the beta-globin gene for beta-thalassemia (sequences provided by Jacques Rochette, INSERM U 925-UPJV, Amiens) and the p53 and APC (adenomatous polyposis coli) genes for cancers [20], [21]. The stop codon present in the mouse *mdx* gene is denoted “MDX”. “STOP LAM” is the natural termination codon of laminin and “STOP PLATI” is the mouse platinum coat color mutation. Nonsense mutations are named according to the position of the modified amino acid in the protein sequence.

For each sequence, the stop codon and the surrounding nucleotide context, shown in Table S1, were inserted into the dual reporter vector pAC99 [22]. Readthrough levels were quantified in NIH3T3 cells transiently transfected with the dual reporter vector, in the presence or absence of gentamicin. Some of these nonsense mutations have already been tested in previous studies in our laboratory. However, as variability is commonly observed between batches of gentamicin [23], we test all 66 stop sequences with the same gentamicin preparation (see Materials and Methods).

Readthrough rates ranged from 0.01% (DMD 2726, beta 43, APC 1131) to 0.52% (CF 122) for basal readthrough (B), and from 0.04% (p53 327) to 2.79% (p53 213) in the presence of 800 µg/ml gentamicin (G) (Figure 1, Table S1). The gentamicin response is defined as the factor of increase (I) between basal and gentamicin-induced readthrough levels. This factor of increase varied from 1.6 (DMD 2125) to 16.3 (APC 1131). Considerable variability for the three variables was observed, as previously described. We characterized readthrough levels and the gentamicin response in mammalian cells in more detail, by carrying out statistical analysis.

### Descriptive statistics for basal and gentamicin-induced readthrough levels and for the gentamicin response

We studied the distribution and characteristics of the variables B, G and I, by descriptive statistical analysis (Table S2). For B, the mean was 0.07% and the median was 0.04%; for G, the mean was 0.37% and the median was 0.23%; for I, the mean was 6.04 and the median was 5.46. The difference between the mean and the median indicates asymmetry in the distribution.

**Figure 2:**
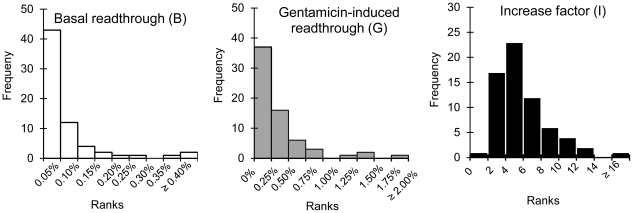
Graphical representations of the distribution of values for the parameters B, G, and I. For each parameter, values are classified into intervals of equal size. X-values indicate the limits for intervals. Y-values indicate the number of values in each interval.

We also carried out a graphical analysis to visualize the distribution of each variable (Figure 2). The variables B and G had a very high kurtosis (flattening coefficient; 11.11 and 12.71, respectively), indicating a sharper peak than for a Gaussian distribution (kurtosis = 0). The asymmetry coefficient was 3.22 for variable B and 3.23 for G, respectively, indicating a strongly asymmetric and L-shaped distribution, with a high proportion of low values. The values were found to be homogeneously distributed, with most located in the first two intervals (Figure 2):

For basal readthrough (variable B), 83.3% of nonsense mutations displayed readthrough levels of no more than 0.10%. Most of the nucleotide contexts surrounding nonsense mutations promoted efficient termination. Readthrough rates above 0.10% accounted for 16.7% of all values and were defined as high basal readthrough levels (see Materials and Methods).
For gentamicin-induced readthrough (variable G), 80.3% of nonsense mutations displayed readthrough rates no higher than 0.5%. Therefore, even in the presence of gentamicin, very few nucleotide contexts promoted “high” levels of readthrough. Readthrough rates above 0.50% accounted for 19.7% of all values and were defined as “high” levels of induced readthrough.

For increase factor (variable I), we observed a kurtosis slightly higher (1.63) than expected for a Gaussian distribution. Its asymmetry coefficient (1.17) was similar to that for a Gaussian distribution ( = 1). The values were homogeneously distributed and 78% of the values had ranks between 4 and 8. Values greater than 8 accounted for 19.7% of all values and were defined as a “high” factor of increase.

We then investigated whether these three distributions could be converted to Gaussian distributions using the Box-Cox transformation (λ = −0.217) (see Materials and Methods), which would make it possible to use more powerful parametric statistics. After transformation, a Shapiro-Wilk test allowed us to conclude that B, G and I variables indeed followed a normal distribution (Figure S1, Table S3).

### Correlation among basal readthrough, induced readthrough, and gentamicin response

**Figure 3:**
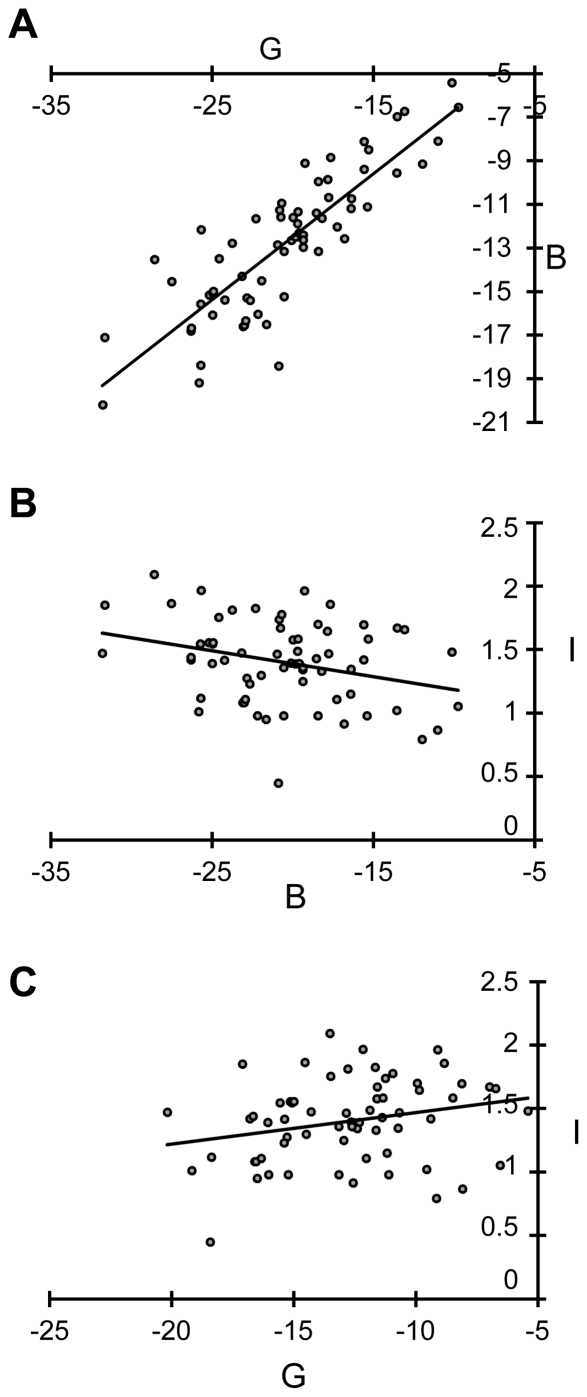
Graphic representations of correlation between the variables B, G, and I after Box-Cox transformation. Gentamicin-induced readthrough level is plotted against basal readthrough level (A); the factor of increase is plotted against basal readthrough level (B) and against gentamicin-induced readthrough level (C). Bravais-Pearson tests were performed to analyze the correlations between the three variables after Box-Cox transformation. Statistically significant results are indicated in Table S4.

Previous observations have suggested that there is no correlation between the basal readthrough level for a nonsense mutation and its sensitivity to gentamicin treatment [3]. However, the sets of mutations analyzed to date have been too small to demonstrate this point statistically. We plotted the level of gentamicin-induced readthrough against basal readthrough and the increase factor against basal readthrough or against gentamicin-induced readthrough before (Figure S2) and after Box-Cox transformation (Figure 3). We used the parametric Bravais-Pearson correlation test to establish the statistical significance of these correlations (Table S4). There was a strong positive correlation between basal readthrough level and gentamicin-induced readthrough level (R = 0.845) and this correlation was significant (p<0.0001). There was a weak negative correlation between basal readthrough and the factor of increase (R = −0.29, p = 0.016), indicative of a trend, with nonsense mutations with a “high” basal readthrough level tending to be less responsive to gentamicin treatment. There was a weak positive correlation between gentamicin-induced readthrough level and the factor of increase (R = 0.25 p = 0.045). Thus, nonsense mutations with ‘high” gentamicin-induced readthrough levels also tended to have the highest factor of increase.

These results provide the first description of the relationship between basal readthrough, gentamicin-induced readthrough and gentamicin response. They indicate that nonsense mutations with a “high” basal readthrough level give “high” levels of gentamicin-induced readthrough. However, some nonsense mutations with a “low” basal readthrough level presented a “high” gentamicin-induced readthrough level, because they had high factors of increase. Thus, nonsense mutations were found to behave in different ways and could be classified into three distinct groups (Table S1):

Response-type 1: High basal readthrough levels and high gentamicin-induced readthrough levels (for example, p53 213, CF 122 or DMD931). These nonsense mutations did not have a particularly high factor of increase.

Response-type 2: A low or medium basal readthrough level associated with high factor of increase, resulting in essentially high levels of gentamicin-induced readthrough (for example, APC 1114 or p53 192).

Response-type 3: A low or medium basal readthrough level and a weak or moderate gentamicin response. Most of the nonsense mutations studied was of this type.

The first two groups include mutations for which gentamicin treatment can promote high levels of readthrough. For these mutations, we would expect to observe clinical benefit for the treatment, with gentamicin, of diseases linked to the presence of a nonsense mutation. Indeed, similar levels of induced readthrough have already been shown to improve the clinical status of cystic fibrosis patients with CFTR mutations treated with gentamicin [19].

We hypothesized that the differences in the behavior of these nonsense mutations should depend on the nature of the stop codon and the nucleotide context. We therefore assessed the contribution of each of these factors.

### Effect of the type of stop codon

**Figure 4:**
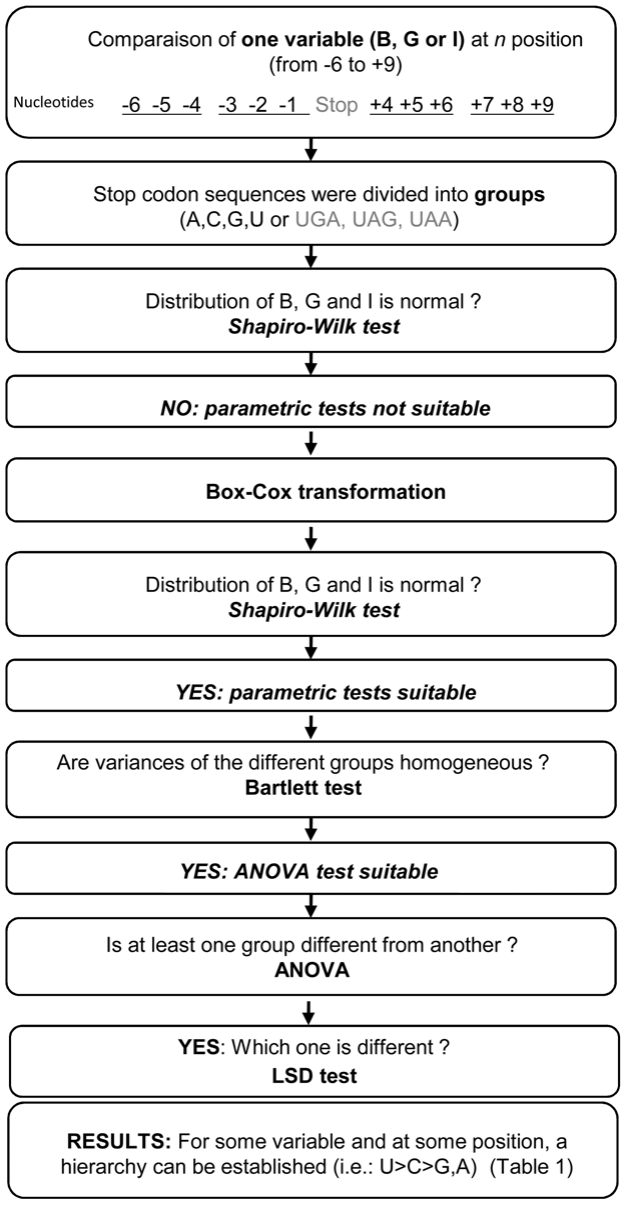
Statistical approach. Description of the statistical approach used for the study of the impact of identity of the stop codon or nucleotide context on readthrough levels and response to gentamicin.

**Figure 5:**
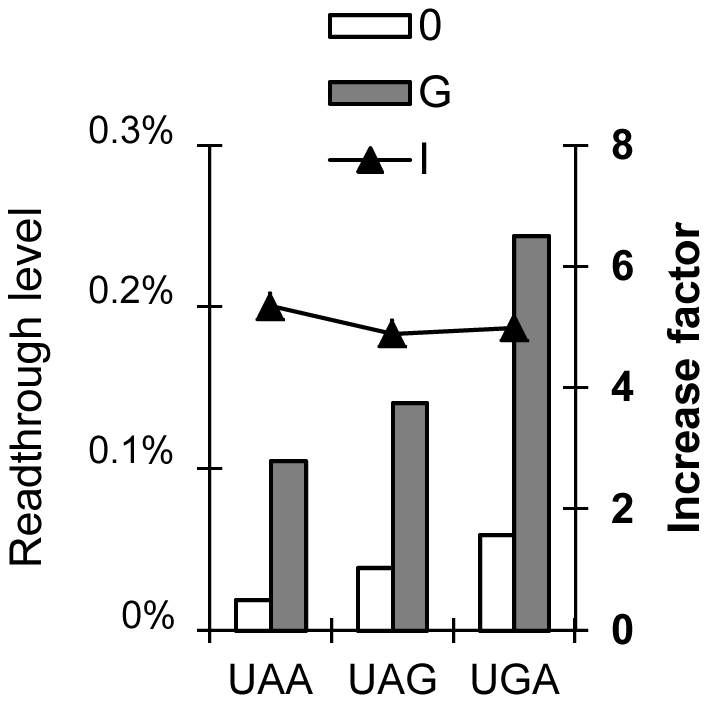
Effect of stop codon identity on readthrough levels and the factor of increase. Graphical representation of medians (before Box-Cox transformation) for the parameters B, G and I are shown for the three different stop codons: UAA, UAG and UGA. The results of the statistical analysis after Box-Cox transformation are shown in Table S5. For basal and gentamicin-induced readthrough levels, the following hierarchy of readthrough level was established: UGA>UAG>UAA. (with>indicating a significant difference). For the factor of increase, UGA = UAG = UAA.

The statistical approach used is described in the Materials and Methods section and in Figure 4. These 66 sequences were assigned to three different groups, according to the nature of the stop codon. There were 14 UAA, 25 UAG and 27 UGA stop codons. The medians of the three variables are shown, for each group, in Figure 5.

After Box-Cox transformation which allowed us to obtain a normal distribution for these 3 variables, an ANOVA test revealed that the three groups differed significantly in terms of their basal and induced readthrough levels. A LSD test yielded the following hierarchy: UGA>UAG>UAA (the sign>represents a statistical difference) for both basal and induced readthrough levels (Table 1 and Table S5). This hierarchy is consistent with previous reports but, to our knowledge, this study provides the first evidence of a statistically significant difference between stop codons. However, some UAA codons have higher readthrough levels than some UGA or UAG codons (i.e. CF 122), highlighting the crucial role of nucleotide context in determining readthrough level in the presence or absence of gentamicin.

**Table 1:** Effects of the nucleotide context and the stop codon on basal readthrough (B), gentamicin-induced readthrough (G) and increase factor (I).

	B	G	I
**−6**	C>U- A>U	/	U>G
**−5**	/	A, C>G	/
**−4**	/	/	/
**−3**	/	/	**A, C, U>G** *
**−2**	/	/	C>A
**−1**	/	**U>A, C, G**	**U>C>A, G** *
**Stop**	**UGA>UAG>UAA** *	**UGA>UAG>UAA** *	/
**+4**	C>A, G, U	**C>A, G, U** *	C>U
**+5**	U>A	/	**A>U, G** *
**+6**	/	/	/
**+7**	/	/	/
**+8**	/	G>C	/
**+9**	/	/	**U>C, G**

Hierarchies were established based on ANOVA/LSD tests after normalization of B, G and I. The sign>indicates a higher level of readthrough or factor of increase. In bold results obtained with a p≤0.05 (Table S5 and Table S6).

*: p≤0.005.

Conversely, ANOVA test revealed that the factor of increase did not differ significantly between the three groups (Table S5). We show here that the factor of increase, which reflects the capacity of a nonsense mutation to respond to treatment, was independent of the nature of the stop codon. The factor of increase therefore probably depends only on the nucleotide context of a given nonsense mutation.

### Effects of nucleotide context

**Figure 6:**
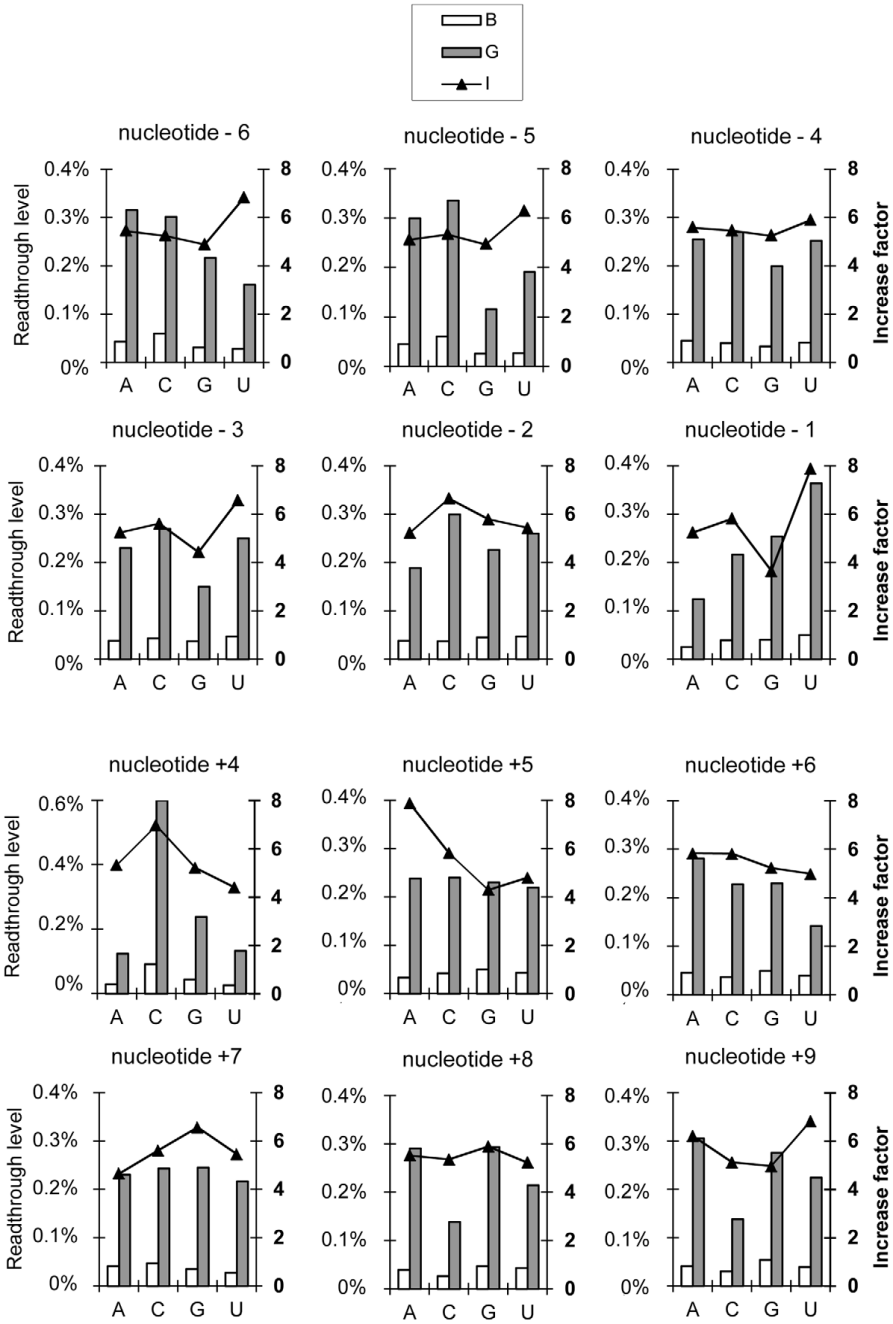
Effect of nucleotide identity at each position (−6 to +9) on readthrough levels and the factor of increase. Graphical representations of medians for the parameters B, G and I (before Box-Cox transformation) for the four different nucleotides (A, C, G or U) at each position in the sequence surrounding the codon stop (−6 to +9). Statistical analysis was performed after Box-Cox transformation and results are summarized in Table 1 and detailed in Table S6.

We then investigated the effects of nucleotide context, by the same statistical approach used for investigation of the effects of stop codon identity (see Materials and Methods and Figure 4). Six nucleotides upstream and downstream from the stop codon have already been shown to influence readthrough level in eukaryotes [6]. We therefore analyzed the effect of each nucleotide in this interval. Graphic representations of the medians of B, G and I (before normalization of the data), for each nucleotide, at each position (from −6 to +9), were generated (Figure 6).

After Box-Cox transformation we were able to use parametric statistical tests (ANOVA and LSD) to define a hierarchy for some positions (Table 1). Bartlett p value and ANOVA F and p-values are indicated in Table S6, Table S7 and Table S8. These analyses were conducted with the complete data set, but could not be applied to each class of stop codon separately, because the number of mutations in each class was too small for statistical analysis. Our analysis thus only identified determinants valid for all three types of stop codon.

We first compared the effect of each nucleotide at a given position to the three others at the same position (Table S6). Our findings confirmed the involvement of distal 5′ and 3′ determinants of nucleotide sequence context in the control of readthrough level and gentamicin response. Indeed, for the nucleotides in positions −6 to +9, we were able to establish correlations between particular classified bases and high levels of readthrough or strong gentamicin response. Nucleotides can be classified according to their effect, for at least one variable, for nine (−6, −5, −3, −2, −1, +4, +5, +8, +9) of the twelve positions studied. For example, an adenine or a cytosine residue in position −6 was associated with higher basal readthrough levels than observed for a uracil residue. A guanine residue in position +8 was associated with a stronger gentamicin-induced readthrough than a cytosine residue in this position. The presence of a uracil residue in position +9 was associated with a stronger gentamicin response than a cytosine or a guanosine residue in this position (Table 1).

These findings contrast with results previously obtained in yeast, which pointed out the role of the two adenine residues immediately upstream from the stop codon in the absence of treatment [8]. This discrepancy is possibly due to differences between mammals and yeast, or to the use of a sequence harboring a motif downstream from the stop codon responsible for promoting particularly high levels of readthrough in this previous study.

Two major determinants were identified in this study (Table 1):

The +4 position, at which a cytosine residue is correlated with higher basal (p = 0.06) and gentamicin-induced readthrough (p = 0.001) than the tree other nucleotides. A C residue in this position has been shown to promote high levels of readthrough in both yeast and mammalian cells, but only in a small number of nucleotide contexts [4], [24]. This statistical analysis demonstrates that this effect operates for a large set of sequences.
The −1 position, at which a uracil residue is associated with higher levels of gentamicin-induced readthrough level (p = 0.02) and a stronger gentamicin response (p<0.005) than for other nucleotides. This is the first time that the presence of a nucleotide at a specific position has been linked to a better response to gentamicin treatment.

However among mutation presenting a U in −1 position, 30% also present a C in +4 (against 8% for mutation with an A, 12% for mutation with a G and 29% for mutation with a C in −1). To assess that the effect of U in −1 is not biased by the presence of a C in +4, we performed the same statistical analysis restricting the pool of mutations to those without a C in +4. In this subset, the mean value of gentamicin-induced readthrough and increase factor is even better when there is a U in −1 position compared to the 3 other nucleotides (Table S7). This result confirms the effect of a uracil in −1 position independently of the presence of a C in +4. Nevertheless, according to the statistical test performed, it can be noticed that for induced-readthrough level there is a combined effect between the U in −1 position with a C in +4 position. Indeed, all the nonsense mutations studied that carried a U Stop C sequence systematically displayed readthrough levels exceeding 0.5% in the presence of gentamicin.

We checked that the determinants identified here were retrospectively consistent with published readthrough analyses. Keeling and Bedwell [5] measured the levels of readthrough induced by several aminoglycosides in a mammalian translation system. The mutation displaying the highest levels of gentamicin- and amikacin-induced readthrough was indeed the only one with a “U stop C” sequence. The combination of these two nucleotides on either side of the stop codon therefore constitutes the first rule ever elucidated for identifying patients with nonsense mutations likely to respond to aminoglycoside treatment.

We also compared the effect of each nucleotide at a given position to the four nucleotides at all positions on B, G and I. This procedure reveal a clear effect for the increase factor p = 0.0002 (Table S8). For example a uracil residue in position −1 was associated with higher increase factor than observed for a guanosine residue in position −3.

### Experimental validation of the effect of the nucleotide in position −1

We analyzed the effect of the nucleotide in position −1 independently of the influence of other nucleotides, by quantifying the readthrough levels of six nonsense mutations in which we changed only the nucleotide in position −1, keeping the rest of the sequence constant:

DMD 673 (UAG, response-type 1) and DMD 319 (UGA, response-type 1) have a U residue in position −1, which we replaced with each of the other three nucleotides (A, C, G) separately.
CF 122 (UAA, response-type 1), DMD 931 (UAG, response-type 1), beta 17 (UAG, response-type 3) and p53 146 (UGA, response-type 3) have a G, A, C or G residue, respectively, in position −1. We replaced each of these residues by a U residue.

**Figure 7:**
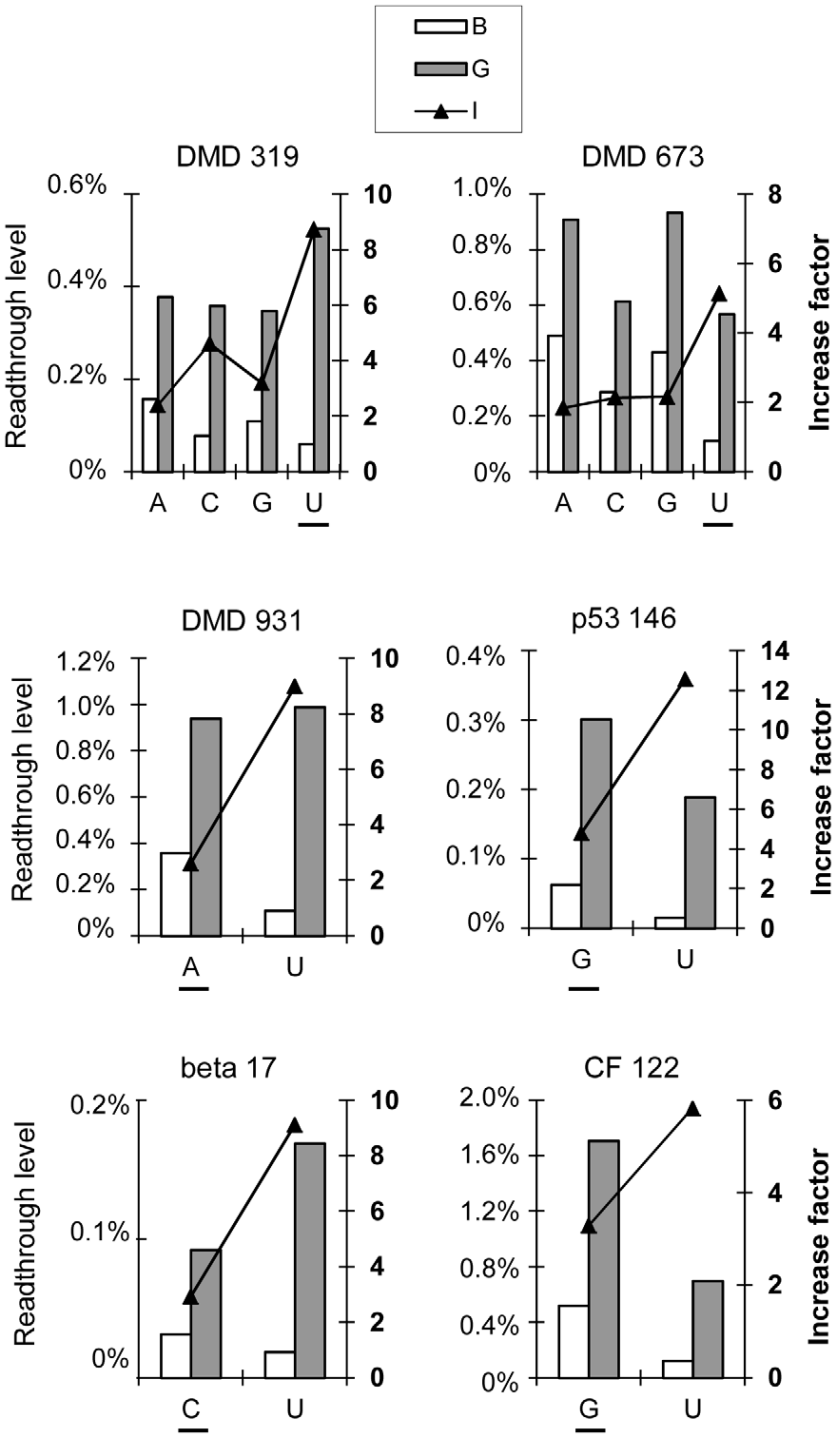
Effect of the nucleotide upstream from the stop codon (−1) on readthrough levels and response to gentamicin. For DMD 319 and DMD 673, a U residue immediately upstream from the stop codon (−1) was replaced by each of the other nucleotides, separately. For DMD 931, p53 146, beta 17 and CF 122, the original nucleotide in the −1 position was replaced by a U residue. Readthrough efficiencies were measured as described in Figure 1. Each value shown is the mean of at least five independent experiments. The original nucleotide is underlined on each graph.

Readthrough levels were quantified in NIH3T3 cells in the presence or absence of gentamicin (Figure 7), statistical data and standard error of the mean are indicated in Table S9. We found that the presence of a U residue in the −1 position was systematically associated with a higher factor of increase than the presence of any other nucleotide in this position. This result confirms the statistical analysis of the 66 nonsense mutations. However, for this narrow panel of stop contexts, gentamicin-induced readthrough levels were not necessarily higher in the presence of a U residue. These levels could be lower (DMD 673, p53 146 and CF 122), equivalent (DMD 931) or higher (DMD 319, beta 17). The effect of the nucleotide in position −1 on gentamicin-induced readthrough level therefore depends strongly on the nature of the other nucleotides surrounding the stop codon.

These results provide evidence that the nucleotide immediately upstream from the stop codon is a major determinant of gentamicin response, with an uracil residue in this position associated with stronger responses to gentamicin treatment. For therapeutic purpose, readthrough levels in presence of gentamicin are the relevant variable. However, the capacity of a nonsense mutation to increase its readthrough level after antibiotic treatment could be a crucial point in the future as new readthrough inducers will be available. Indeed several groups are currently developing news molecules derived from aminoglycosides and acting in a similar way but with a greater efficiency [25]–[27]. In this case, a nonsense mutation with a good increase factor could overtake the threshold of 0.5% of readthrough.

We then examined how the nucleotide upstream from the stop codon exerted its effect on readthrough levels. In prokaryotes, the chemical properties of the ultimate amino acid in the nascent polypeptide chain have been reported to modulate translational readthrough [28], [29]. The −1 nucleotide may also influence readthrough by interacting directly with the P site tRNA or indirectly with eRF1. We therefore investigated whether the final tRNA or amino acid incorporated had an effect on readthrough levels.

### The nature of the last amino acid has no effect on readthrough levels

The nucleotide in the −1 position is the third base of the codon immediately upstream from the stop codon (codon −1). During translation termination, the stop codon is located in the ribosomal A-site and codon −1 is in the P-site. We therefore investigated whether having a hydrophilic or hydrophobic amino acid at the P site was correlated with higher levels of readthrough or stronger gentamicin responses. A two-tailed t-test comparing the two groups (hydrophobic or hydrophilic amino acid) for all the nonsense mutations studied showed that there is no relationship between the nature of the final amino acid and a high factor of increase (t = −0.91; p = 0.36) or high readthrough rates (t = 1.71; p = 0.09 for B and t = 1.28; p = 0.2 for G). Moreover, the amino acids encoded by codons ending in U do not belong to a particular chemical class.

These results strongly suggest that the nature of the amino acid at the ribosomal P-site is not a major determinant of readthrough levels.

### The identity of the tRNA at the P site has no effect on readthrough levels

We then investigated whether the effect of the nucleotide in the −1 position on the factor of increase was due to the nature of the tRNA at the P site. Nucleotides 1, 2 and 3 of the mRNA codon are recognized by nucleotides 36, 35 and 34, respectively of the tRNA anticodon (Figure S3A). Codons ending in a C or U residue may be recognized through wobble pairing at position 34 of the anticodon (Figure S3B). In such situations, a single tRNA may recognize several codons. There are two possibilities in eukaryotes: nucleotides U3 and C3 of the codon may be recognized by nucleotides A34 or G34 on the anticodon. Thus, U3 may be recognized by wobble pairing (G34) or Watson-Crick pairing (A34). We investigated the way in which recognition of the codon in position −1 affected readthrough levels or gentamicin response, by comparing nonsense mutations for which the −1 codon is recognized by wobble pairing with those recognized by Watson-Crick pairing, in two-tailed t-test. We found no significant difference between these two types of nonsense mutation, for B (t = 0.61; p = 0.54), G (t = 0.63; p = 0.53) or I (t = −0.099; p = 0.92). The strength of base pairing between the −1 codon and the corresponding anticodon therefore seems to have no influence on readthrough levels or gentamicin response.

**Figure 8:**
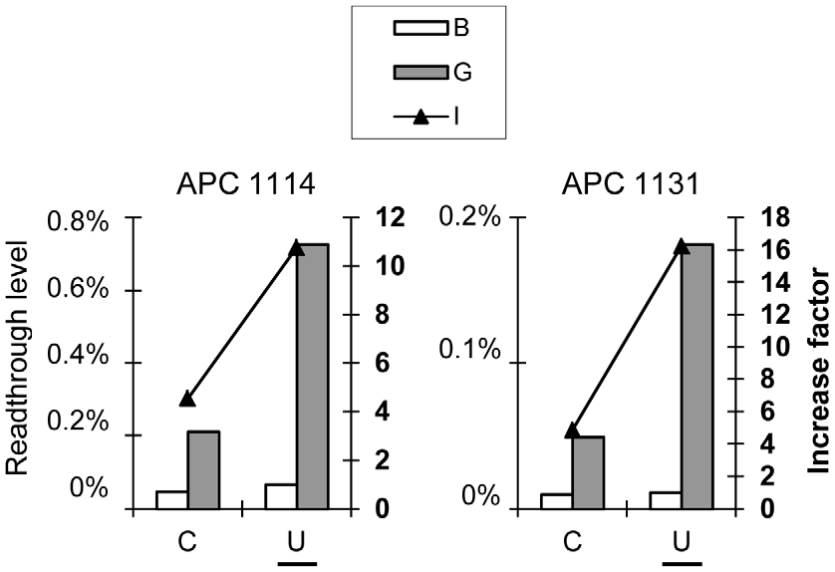
The nature of the tRNA at the P site is not the major determinant of the gentamicin response. The APC 1114 and APC 1131 nonsense mutations originally had a U residue in the −1 position, which we replaced with a C residue. For each sequence, the two different codons at the P-site are recognized by the same tRNA (Table 2). Readthrough efficiencies were measured as described in Figure 1. Each value shown is the mean of at least five independent experiments. The original nucleotide is underlined on each graph.

We investigated whether the effect of the −1 nucleotide on the factor of increase was correlated with the identity of the tRNA, using four nonsense mutations: beta 17, DMD 319, (Figure 7) and APC 1131 (UAA, response-type 2), APC 1114 (UGA, response-type 2) (Figure 8). The third base of the −1 codon of these nonsense mutations was changed to create an alternative –1 codon recognized by the same tRNA (Table 2). For example, the AAU −1 codon of the APC 1114 nonsense mutation was replaced by an AAC codon, which is also recognized by the (3′→5′) UUG anticodon of the same tRNA ASN. Readthrough levels were quantified in NIH3T3 cells transiently transfected with the dual reporter vector containing the appropriate sequence, in the presence or absence of gentamicin. For these nonsense mutations, the factor of increase and the gentamicin-induced readthrough were higher when there was a U residue in position −1 than when there was another base in this position, while the modified codon was recognized by the same tRNA. These findings provide strong evidence for a lack of involvement of the tRNA at the ribosomal P-site in determining the gentamicin response.

**Table 2:** Codon and anticodon immediately upstream of the stop codon (−1 position).

Nonsense mutations	Beta 17		DMD 319		APC 1114		APC 1131	
Stop codon	UAG		UGA		UGA		UAA	
Nucleotide in −1	C	U	C	U	C	U	C	U
Last Amino-acid	GLY	GLY	PRO	PRO	ASN	ASN	CYS	CYS
Codon (5′→3′) in −1	GGC	GG**U**	CC**C**	CCU	AA**C**	AAU	UGC	UG**U**
Anticodon (3′→5′)	CCG	CC**G**	GG**A**	GGA	UU**G**	UUG	ACG	AC**G**

Codons at −1 of these sequences are recognized by the same tRNA. In bold, the original nucleotide at −1 position and the Wobble pairing.

Thus, the nucleotide in the −1 position is itself a major determinant of the gentamicin response and of the gentamicin-induced readthrough level.

### Conclusion

We used the largest set of nonsense mutations ever analyzed for the first statistical analysis of the influence of nucleotide context on PTC readthrough and response to aminoglycoside treatment. We confirm the findings of previous studies concerning the importance of the nucleotide in the +4 position, at which the presence of a cytosine (C) residue is correlated with high basal and gentamicin-induced readthrough levels. We also show for the first time that the presence of a U residue in −1 is a key determinant of gentamicin-induced readthrough which is the relevant parameter for clinical applications. Moreover, we can notice that a U in −1 is also correlated with a higher increase factor between basal and gentamicin-induced readthrough. This finding may have important implications in fundamental aspects of structural interactions between readthrough inducers and the translational apparatus. We show that impact of the base in the −1 position is mediated neither by the last amino acid nor by the tRNA present at the ribosomal P site.

These data are consistent with previous reports excluding a role for the last residue of the polypeptide chain or the last incorporated tRNA in readthrough efficiency in eukaryotes [8], [30]. Different rules seem to apply in prokaryotes, as the two last amino acids and the tRNA present in the P site have been shown to influence termination efficiency in *E. coli* [31]. It remains unclear how this nucleotide modulates the factor of increase in mammals. One possible hypothesis is that the stacking properties of this base in the vicinity of the stop codon are involved in the balance between translation termination and suppression. More generally, this nucleotide, which is close to the decoding center targeted by aminoglycosides, may induce local structural rearrangements favoring the influence of aminoglycosides at the ribosomal A site.

Finally, the consensus sequence U STOP C was systematically associated with induced readthrough levels greater than 0.5%. The combination of these two nucleotides before and after the stop codon may therefore provide an initial indicator of readthrough levels compatible with therapeutic benefit.

## Materials and Methods

### Cell lines and cell culture

NIH3T3 cells (embryonic mouse fibroblasts kindly provided by Marc Sitbon) were cultured in DMEM plus GlutaMAX (Invitrogen). The medium was supplemented with 10% foetal calf serum (FCS, Invitrogen) and 100 U/ml penicillin/streptomycin. Cells were kept in a humidified atmosphere containing 5.5% CO_2_ at 37°C.

### Readthrough quantification in cell culture

Complementary oligonucleotides corresponding to nonsense mutations embedded in their natural context (sequences in Table S1) were annealed and ligated into the pAC99 dual reporter plasmid, as previously described [22]. This dual reporter can be used to quantify stop-codon readthrough, through the measurement of luciferase and beta-galactosidase (internal calibration) activities, as previously described [19]. Readthrough levels for nonsense mutations were analyzed in the presence or absence of gentamicin. All nonsense mutations were tested with batches of gentamicin with identical efficiency of readthrough levels. For p53 and APC, results were obtained from recent studies [20], [21]. For CF, DMD and CMD [19], [3] and [16], readthrough levels had already been estimated but the tests were repeated in this study, to prevent discrepancies due to the use of different batches of gentamicin. NIH3T3 cells were electroporated with 20 µg of reporter plasmid. The following day, the cells were rinsed and fresh medium, with or without gentamicin (800 µg/ml), was added. No cell toxicity was observed with this dose of antibiotic. Cells were harvested 24 hours later, by trypsin-EDTA treatment (Invitrogen) and lysed. Beta-galactosidase and luciferase activities were assayed as previously described [22]. Readthrough efficiency was estimated by calculating the ratio of luciferase activity to beta-galactosidase activity obtained with the test construct, normalizing the value obtained with respect to that obtained with an in-frame control construct. For each construct, we performed at least three independent transfection experiments (3 to 10 experiments).

### Statistical analysis

Excel was used for statistical analysis: the Analysis Toolpack for descriptive statistics; the XL- stat for Bartlett correlation tests (Bravais-Pearson) and t-tests, Analyse-it module for ANOVA and LSD tests.

Descriptive statistics (Table S2) provided simple information (mean, median etc.) about three variables: basal readthrough level (B), gentamicin-induced readthrough level (G) and the factor of increase between basal and induced readthrough levels (I). The median, which is obtained by arranging the values in size order and selecting the middle value, is useful when the distribution does not follow a Gaussian distribution (i.e. for variables that do not tend to cluster around a single mean value). Graphical analysis was performed and the values of each variable were ranked (Figure 2). The intervals between different ranks were identical and no minimal number of values per rank was required. The intervals and number of rows were defined according to a convention taking into account the total number of values and the minimal and maximal values for each of the variables studied. Values included in ranks corresponding to the best ∼20% of values were defined as “high”.

Several parameters (Kurtosis coefficient, asymmetry coefficient etc.) and graphical analysis were used to determine whether the distribution of each variable followed a Gaussian distribution. A Gaussian distribution is characterized by a Kurtosis coefficient of 0 and an asymmetry coefficient of 1.

In order to perform a complete statistical analysis we chose to use parametric tests instead of low-power non-parametric analysis. To this aim, we performed a Box-Cox transformation for variables B, G and I with Ψ_λ_(x) = (x^λ^−1)/λ using the same lambda: −0.217. After this transformation, a Shapiro-Wilk test allowed us to conclude that B, G and I follow a normal distribution.

Correlations between variables were analyzed with the parametric Bravais-Pearson test. The null hypothesis (H_0_) was “there is no correlation between the two variables studied (R = 0). The alternative hypothesis (H_1_) was “there is a correlation between the two variables studied (R≠0)”. A perfect positive correlation gives an R value of +1, whereas a perfect negative correlation gives an R value of −1. The significance level was set at 0.05.

In order to analyze the effect of the nature of the stop codon or the nucleotide context on readthrough levels and gentamicin response the 66 stop codons were divided into three groups for stop codon studies (UAA, UAG and UGA) and four groups for nucleotide context studies (U, C, A, G). For each group, we then used a Bartlett test to analyze heterogeneity of variance of each variable. If heterogeneity was not significant we performed one of the most commonly used multiple comparison procedure, the Fisher's Least Significant Difference (LSD) test. The LSD test is a two-step test. First an ANOVA (Analysis Of Variance) test is performed. The null hypothesis for ANOVA is that the mean (average value of the dependent variable) is the same for all groups. The alternative hypothesis is that the mean is not the same for all groups. When the null hypothesis is rejected, it means that at least 2 groups are different from each other. In a second step we determine which groups are different from which performing all pairwise t-tests. This last procedure allows to establish a hierarchy between stop or nucleotide at each position. In Table S6 each nucleotide at a given position is compared to the others at the same position and in Table S8 each nucleotide is compared to the four nucleotides at all position.

Two-tailed Student's t-tests (excel) were used to study the influence of tRNA or the amino acid in the ribosomal P-site. For this test, we used a significance level α of 0.05.

## Supporting Information

Figure S1 Plots representing normal distribution of B, G and I after Box-Cox transformation using a common lambda = −0.217.
(TIF)

Figure S2 Graphic representations of correlation between the variables B, G and I before Box-Cox transformation. Gentamicin-induced readthrough level is plotted against basal readthrough level (A); the factor of increase is plotted against basal readthrough level (B) and against gentamicin-induced readthrough level (C). For each graph, the trend curve is shown.
(TIF)

Figure S3 Codon-anticodon recognition rules. A. The nucleotides in positions 1, 2 and 3 of the codon are recognized by the nucleotides in positions 36, 35 and 34, respectively, of the tRNA anticodon. B. In eukaryotes, the nucleotide in position 34 of the anticodon can recognize two different nucleotides in position 3 of the codon. A, U or a C residue in position 3 of the codon may be recognized by an A or a G residue in position 35 of the tRNA. Thus, some codons are recognized by wobble pairing with the anticodon.
(TIF)

Table S1 List of the 66 sequences containing a stop codon, with basal readthrough (B), gentamicin induced readthrough (G), increase factor between basal and induced readthrough (I) and the classified group in response-type. These sequences were inserted into the dual reporter vector in order to determine readthrough level. Nonsense mutations are named by the gene or the disease related to and by their position (amino-acid). p53 mutations are involved in cancers; DMD and CMD mutations are involved in muscular dystrophies; CF mutations are involved in cystic fibrosis and beta mutations are involved in beta-thalassemia disease (see Materials et Methods for references). Nonsense mutations are classified according to their gentamicin induced readthrough level.
(PDF)

Table S2 Descriptive statistics for the 3 parameters B, G and I before Box-Cox transformation.
(PDF)

Table S3 Shapiro Test after Box-Cox transformation (λ = −0.217).
(PDF)

Table S4 Bravais-Pearson statistical analysis of correlation between basal readthrough, induced readthrough and gentamicin response (Increase Factor) after Box-Cox transformation.
(PDF)

Table S5 Statistical analysis of the effect of the type of stop codon on B, G and I after Box-Cox transformation using a common lambda: −0.217 (this transformation leads to negative value for B and G).
(PDF)

Table S6 Statistical analysis of the effect of each nucleotide at each position on B, G and I after Box-Cox transformation using a common lambda: −0.217 (this transformation leads to negative value for B and G).
(DOC)

Table S7 Statistical analysis of the effect of nucleotide in −1 position on B, G and I (after Box-Cox transformation) restricting the pool of mutations to those without a C in +4.
(DOC)

Table S8 Statistical analysis of the effect of all nucleotides on B, G and I after Box-Cox transformation using a common lambda: −0.217. The significant differences between each nucleotide against all nucleotides at all positions are listed. In this list, inc is for Increase Factor A, G, C or U for the nucleotide and the − or + followed by a number for position.
(DOC)

Table S9 Statistical data (Standard deviation and standard error of the mean) of studied nonsense mutations (Figure 7 and Figure 8).
(DOC)
