# Supplementary figures and images for: Statistical Analysis of Readthrough Levels for Nonsense Mutations in Mammalian Cells Reveals a Major Determinant of Response to Gentamicin
Source: PLoS Genet. 2012 Mar 29;8(3):e1002608. doi: 10.1371/journal.pgen.1002608 (PMC3315467; doi:10.1371/journal.pgen.1002608)

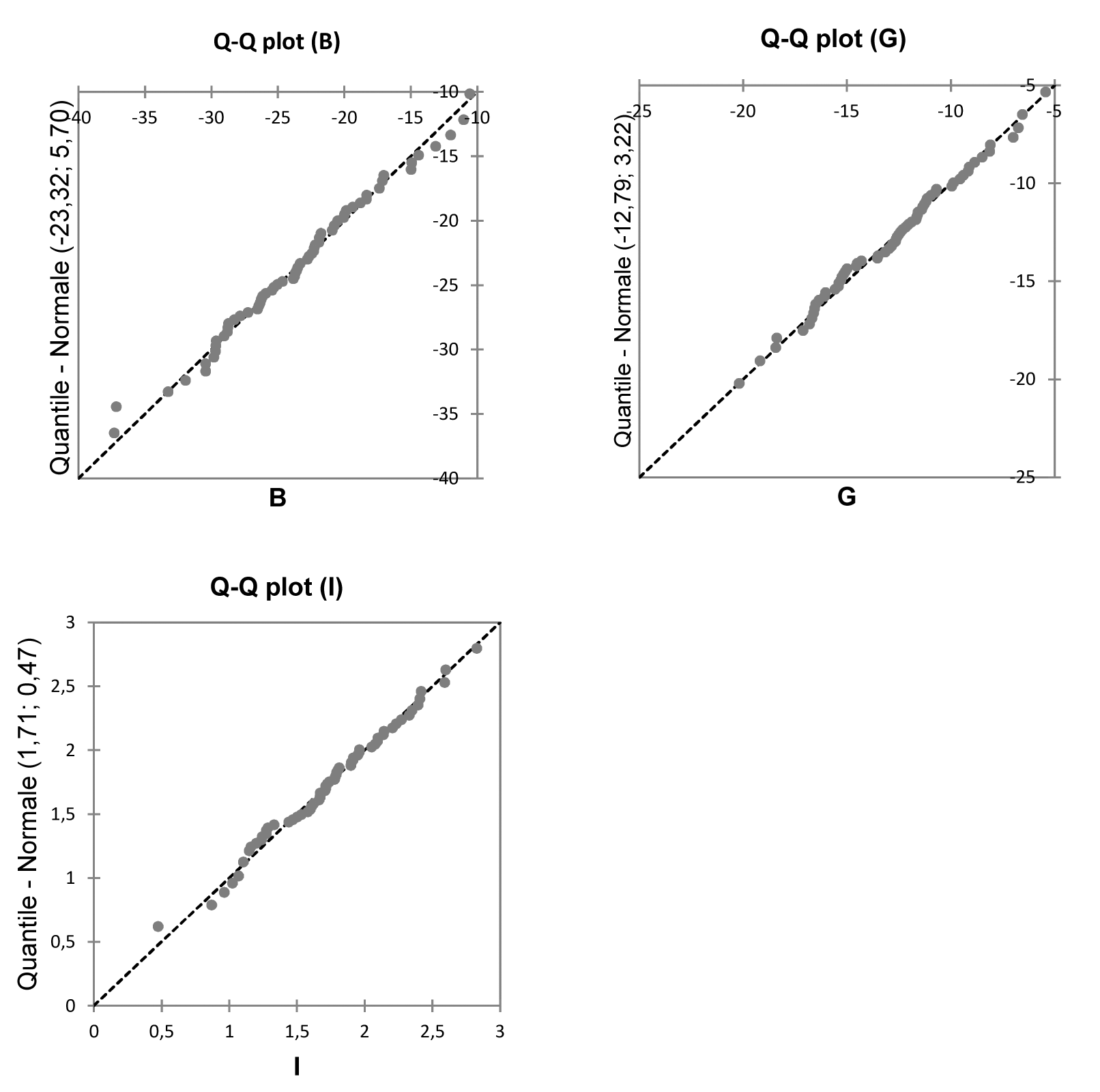

Supplement: Figure S1 — Plots representing normal distribution of B, G and I after Box-Cox transformation using a common lambda = −0.217. (TIF) [file pgen.1002608.s001.tif]

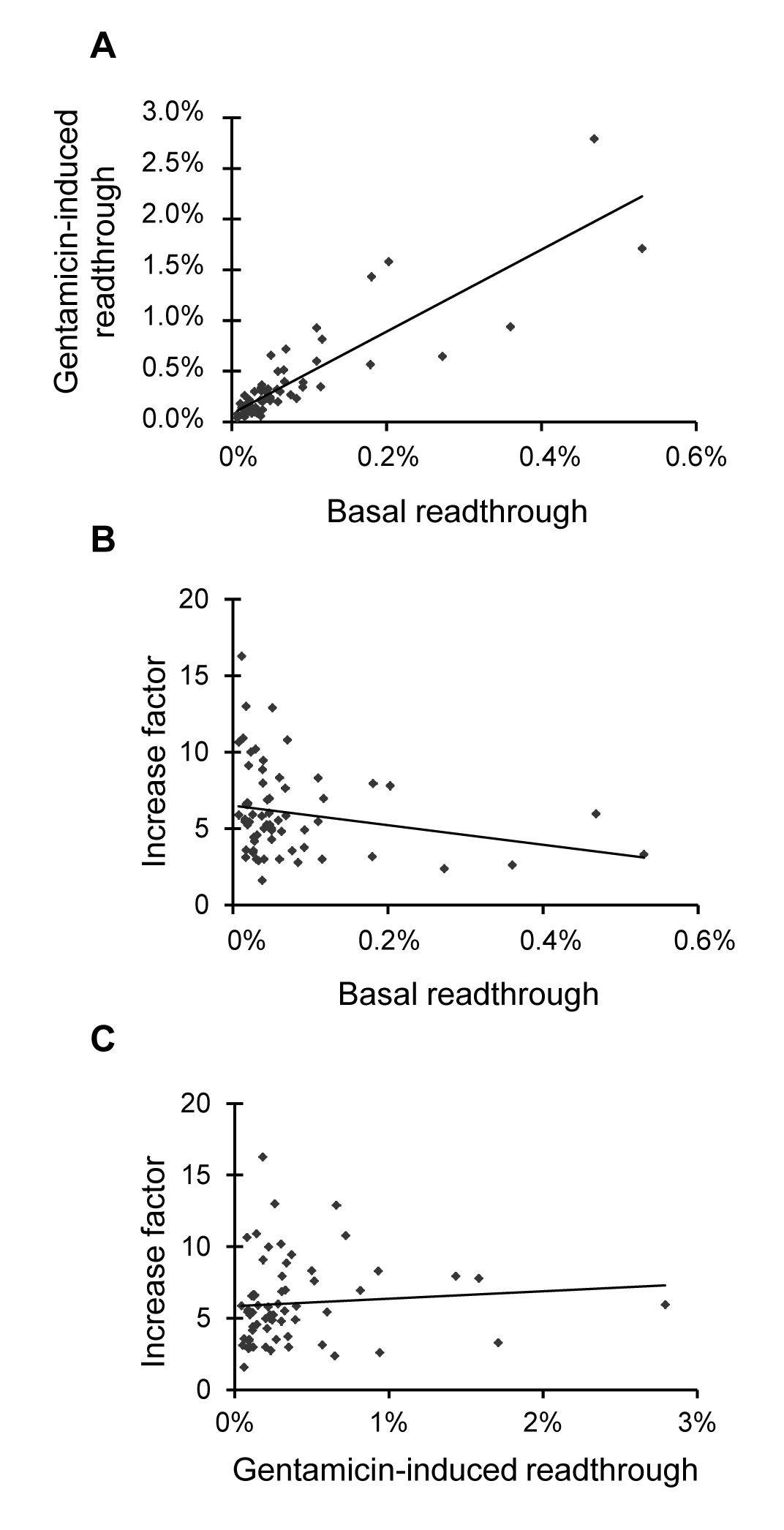

Supplement: Figure S2 — Graphic representations of correlation between the variables B, G and I before Box-Cox transformation. Gentamicin-induced readthrough level is plotted against basal readthrough level (A); the factor of increase is plotted against basal readthrough level (B) and against gentamicin-induced readthrough level (C). For each graph, the trend curve is shown. (TIF) [file pgen.1002608.s002.tif]

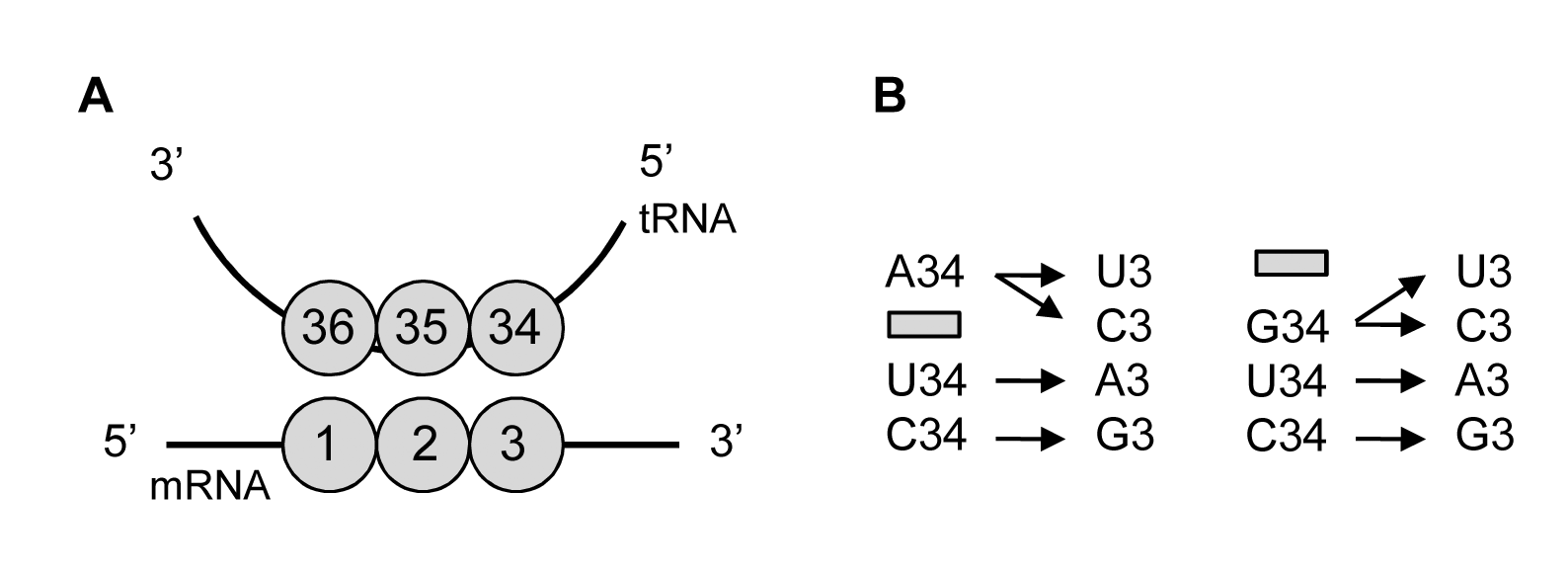

Supplement: Figure S3 — Codon-anticodon recognition rules. A. The nucleotides in positions 1, 2 and 3 of the codon are recognized by the nucleotides in positions 36, 35 and 34, respectively, of the tRNA anticodon. B. In eukaryotes, the nucleotide in position 34 of the anticodon can recognize two different nucleotides in position 3 of the codon. A, U or a C residue in position 3 of the codon may be recognized by an A or a G residue in position 35 of the tRNA. Thus, some codons are recognized by wobble pairing with the anticodon. (TIF) [file pgen.1002608.s003.tif]
