# Supplementary material for: Statistical Analysis of Readthrough Levels for Nonsense Mutations in Mammalian Cells Reveals a Major Determinant of Response to Gentamicin
Source: PLoS Genet. 2012 Mar 29;8(3):e1002608. doi: 10.1371/journal.pgen.1002608 (PMC3315467; doi:10.1371/journal.pgen.1002608)
Supplement: Table S1 — List of the 66 sequences containing a stop codon, with basal readthrough (B), gentamicin induced readthrough (G), increase factor between basal and induced readthrough (I) and the classified group in response-type. These sequences were inserted into the dual reporter vector in order to determine readthrough level. Nonsense mutations are named by the gene or the disease related to and by their position (amino-acid). p53 mutations are involved in cancers; DMD and CMD mutations are involved in muscular dystrophies; CF mutations are involved in cystic fibrosis and beta mutations are involved in beta-thalassemia disease (see Materials et Methods for references). Nonsense mutations are classified according to their gentamicin induced readthrough level. (PDF) [file pgen.1002608.s004.pdf]

Table S1: List of the 66 sequences containing a stop codon, with basal readthrough (B), gentamicin induced readthrough (G), increase factor between basal and induced readthrough (I) and the classified group in response-type.

Stop codon and its nucleotide context [a]																								Name [b]	B	G±	I	Response-Type
						-6	-5	-4	3	-2	-1	Stop			+4	+5	+6	+7	+8	+9								
A	G	A	A	A	C	A	C	T	T	T	T	T	G	A	C	A	T	A	G	T	G	T	G	p53 213	0.47%	2.79%	6,0	1
C	G	C	T	C	T	A	T	C	G	C	G	T	A	A	C	T	A	G	G	C	A	T	A	CF 122	0.52%	1.71%	3.3	1
C	T	C	A	T	C	C	A	G	C	T	T	T	G	A	C	A	T	G	G	C	A	A	T	APC 360	0.20%	1.58%	7.8	1
G	C	C	A	C	A	C	T	G	A	G	T	T	A	G	C	T	G	C	A	C	T	G	T	beta 90	0.18%	1.43%	7.9	1
G	C	C	A	G	A	G	A	G	A	A	A	T	A	G	C	T	A	C	A	G	A	C	A	DMD 931	0.36%	0.94%	2.6	1
A	A	A	A	A	C	A	A	A	T	T	T	T	G	A	A	C	C	A	A	A	A	G	G	DMD 3381	0.11%	0.93%	8.3	1
T	T	C	T	G	C	A	C	G	T	G	C	T	G	A	C	C	T	G	G	A	G	C	C	CMD 1549	0.11%	0.82%	7.5	1
T	C	A	G	A	A	A	C	A	A	A	T	T	G	A	G	T	G	G	G	T	T	C	T	APC 1114	0.07%	0.73%	10.8	2
C	T	G	G	C	C	C	C	T	C	C	T	T	A	G	C	A	T	C	T	T	A	T	C	p53 192	0.05%	0.66%	12.9	2
G	A	C	C	G	A	C	A	A	G	G	G	T	G	A	T	T	T	G	A	C	A	G	A	DMD 2098	0.27%	0.65%	2.4	1
G	T	C	A	C	C	A	C	C	A	C	T	T	A	G	C	C	A	T	C	A	C	T	A	DMD 673	0.11%	0.57%	5.2	1
T	A	T	G	A	T	A	C	G	G	G	A	T	G	A	A	C	A	G	G	G	A	G	G	DMD 3190	0.18%	0.57%	3.2	1
G	A	G	C	C	T	C	A	C	C	A	C	T	A	G	C	T	G	C	C	C	C	C	A	p53 298	0.07%	0.52%	7.6	2
A	G	C	C	C	A	T	T	T	C	C	T	T	G	A	C	A	G	C	A	T	T	T	G	DMD 319	0.06%	0.53%	8.8	2
T	T	T	G	C	T	C	A	G	T	T	T	T	G	A	A	G	A	C	T	A	A	A	C	DMD 1967	0.07%	0.40%	5.8	3
A	C	C	T	G	C	C	C	T	G	T	G	T	A	G	C	T	G	T	G	G	G	T	T	p53 144	0.09%	0.39%	4.9	3
A	G	C	T	C	C	T	C	T	C	C	C	T	A	G	C	C	A	A	A	G	A	A	G	p53 317	0.04%	0.37%	9.5	2
A	C	T	T	T	G	C	A	A	C	A	G	T	G	A	A	G	G	A	A	A	G	C	C	CF 282	0.12%	0.35%	3.0	3
G	T	T	A	C	T	G	C	C	C	T	G	T	G	A	G	G	C	A	A	G	G	T	G	beta 15 TGA	0.09%	0.34%	3.8	3
G	A	T	G	A	T	A	A	T	A	G	G	T	G	A	G	A	C	A	A	T	T	T	T	APC 811	0.04%	0.34%	8.9	2
A	T	C	C	A	A	T	C	T	G	A	T	T	A	G	A	C	A	A	G	T	C	A	T	DMD 1417	0.05%	0.33%	7.0	3
T	T	C	G	A	G	A	T	G	T	T	C	T	G	A	G	A	G	C	T	G	A	A	T	p53 342	0.06%	0.32%	5.5	3
G	C	T	C	A	A	A	C	C	A	A	G	T	G	A	G	A	A	G	T	A	C	C	T	APC 1450	0.04%	0.31%	8.0	2
C	A	G	C	A	T	C	T	T	A	T	C	T	G	A	G	T	G	G	A	A	G	G	A	p53 196	0.04%	0.31%	6.9	3
C	C	T	G	T	G	C	A	G	C	T	G	T	G	A	G	T	T	G	A	T	T	C	C	p53 146	0.06%	0.30%	4.8	3
G	T	G	G	T	C	T	A	C	C	C	T	T	G	A	A	C	C	C	A	G	A	G	G	beta 37TGA	0.03%	0.30%	10.2	2
G	A	T	A	T	G	G	A	A	A	A	A	T	G	A	G	C	A	C	A	G	C	G	A	APC 213	0.05%	0.28%	6.0	3
G	T	T	A	C	T	G	C	C	C	T	G	T	A	G	G	G	C	A	A	G	G	T	G	beta 15 TAG	0.08%	0.27%	3.5	3
A	A	T	A	T	A	G	T	T	C	T	T	T	G	A	G	A	A	G	G	T	G	G	A	CF 542	0.02%	0.26%	13.0	2
A	G	T	A	T	T	C	C	G	T	T	C	T	G	A	G	G	T	T	G	C	A	T	C	CMD 3085	0.05%	0.25%	5.0	3
G	A	A	G	T	T	G	G	T	G	G	T	T	A	G	G	C	C	C	T	G	G	G	C	beta 26	0.05%	0.24%	4.9	3
G	G	G	A	G	C	A	C	T	A	A	G	T	G	A	G	C	A	C	T	G	C	C	C	p53 306	0.05%	0.24%	5.2	3
G	A	T	A	G	C	C	C	T	G	G	A	T	A	A	A	C	C	A	T	G	C	C	A	APC 1429	0.08%	0.23%	2.8	3
A	G	G	C	C	C	T	G	G	A	A	C	T	G	A	G	G	G	G	C	G	T	T	C	STOP LAM	0.05%	0.23%	5.0	3
G	A	T	A	T	T	G	A	A	C	A	A	T	A	G	T	T	C	A	C	T	G	A	A	p53 53	0.04%	0.23%	5.3	3
C	G	A	T	C	T	G	T	G	A	G	C	T	G	A	G	T	C	T	T	T	A	A	G	CF 1162	0.02%	0.22%	10.0	2
G	G	C	T	G	T	G	T	T	C	C	C	T	G	A	A	A	C	T	G	C	A	A	T	CMD 967	0.04%	0.22%	5.8	3
T	T	G	C	C	C	C	T	G	C	G	C	T	A	G	G	G	A	A	T	T	C	T	C	DMD 2264	0.05%	0.21%	4.3	3
C	C	T	A	G	G	C	A	C	A	G	G	T	G	A	G	T	T	A	A	C	G	G	C	CMD 744	0.04%	0.20%	5.0	3
A	A	C	G	T	G	C	T	G	G	T	C	T	G	A	G	T	G	C	T	G	G	C	C	beta 112	0.06%	0.20%	3.0	3
A	G	A	A	C	T	G	T	G	A	C	C	T	G	A	G	A	A	G	A	C	T	T	C	CMD 1326	0.02%	0.18%	9.1	2
C	A	G	T	C	T	T	T	G	T	G	T	T	A	A	G	A	A	G	A	T	G	A	C	APC 1131	0.01%	0.18%	16.3	2
C	T	G	G	A	T	G	G	A	G	A	A	T	A	A	T	T	C	A	C	C	C	T	T	p53 327	0.03%	0.15%	5.9	3
G	T	G	A	A	C	G	T	G	G	A	T	T	A	A	G	T	T	G	G	T	G	G	T	beta E22	0.03%	0.14%	4.6	3
C	A	G	A	G	G	T	T	C	T	T	T	T	A	G	T	C	C	T	T	T	G	G	G	beta 43	0.01%	0.14%	10.9	2
A	A	A	A	G	T	G	G	T	G	C	T	T	A	G	A	C	A	C	C	C	A	A	A	APC 1367	0.02%	0.13%	6.7	3
A	A	A	C	T	T	C	C	A	G	A	A	T	A	A	T	T	T	G	A	A	G	G	A	CMD 1240	0.02%	0.12%	6.6	3
C	A	G	G	A	T	T	T	G	G	A	A	T	A	G	A	G	G	C	G	T	C	C	C	DMD 2522	0.02%	0.12%	6.7	3
A	T	G	G	A	T	A	T	C	C	T	G	T	A	G	A	T	T	A	T	T	A	A	T	DMD 3149	0.04%	0.12%	3.0	3
A	A	C	C	C	T	A	A	G	G	T	G	T	A	G	G	C	T	C	A	T	G	G	C	beta 61	0.03%	0.12%	4.4	3
G	A	T	A	G	A	A	G	T	T	T	G	T	A	G	A	G	A	G	A	A	C	G	C	APC 853	0.02%	0.12%	5.4	3
T	A	C	C	C	T	T	G	G	A	C	C	T	A	G	A	G	G	T	T	C	T	T	T	beta 39	0.03%	0.11%	4.2	3
C	A	A	T	G	T	T	G	G	T	G	C	T	A	A	C	C	T	G	G	A	G	T	C	CMD 988	0.02%	0.11%	6.6	3
A	A	G	A	A	G	A	A	G	A	A	G	T	A	G	A	A	G	C	A	A	C	C	C	STOP PLATI	0.03%	0.10%	3.0	3
G	C	A	G	A	A	A	T	A	A	A	A	T	A	A	A	A	G	A	T	T	G	G	A	APC 1309	0.02%	0.10%	5.2	3
G	C	T	A	C	A	G	A	T	A	T	G	T	A	A	T	T	G	A	C	A	A	A	G	DMD 1593	0.03%	0.09%	3.2	3
G	C	C	C	T	G	T	G	G	G	G	C	T	A	G	G	T	G	A	A	C	G	T	G	beta 17	0.03%	0.09%	2.9	3
C	A	T	C	G	T	A	G	T	A	A	G	T	A	G	A	G	A	C	A	C	A	A	G	APC 789	0.03%	0.09%	3.4	3
C	A	C	T	T	T	G	G	C	A	A	A	T	A	A	T	T	C	A	C	C	C	C	A	beta 121	0.03%	0.09%	3.4	3
G	T	G	G	T	C	T	A	C	C	C	T	T	A	G	A	C	C	C	A	G	A	G	G	beta 37 TAG	0.02%	0.09%	5.6	3
C	T	G	C	T	G	C	T	G	C	T	C	T	A	A	C	C	T	T	G	G	A	C	C	beta 35	0.02%	0.08%	5.5	3
G	A	T	C	A	C	A	T	G	T	G	C	T	A	A	C	A	G	G	T	C	T	A	T	DMD 1143	0.01%	0.08%	10.7	2
T	T	G	A	A	A	G	A	G	C	A	A	T	A	A	A	A	T	G	G	C	T	T	C	MDX	0.02%	0.06%	3.6	3
A	C	A	G	A	A	G	C	T	G	A	A	T	A	G	T	T	T	C	T	C	A	G	A	DMD 2125	0.04%	0.06%	1.6	3
A	C	C	C	C	A	C	C	A	G	T	G	T	A	G	G	C	T	G	C	C	T	A	T	beta 127	0.02%	0.05%	3.1	3
G	A	A	A	G	G	C	T	C	C	T	A	T	A	A	G	A	C	T	C	C	A	A	G	DMD 2726	0.01%	0.04%	5.9	3

[a] These sequences were inserted into the dual reporter vector in order to determine readthrough level.

[b] Nonsense mutations are named by the gene or the disease related to and by their position (amino-acid). p53 mutations are involved in cancers; DMD and CMD mutations are involved in muscular dystrophies; CF mutations are involved in cystic fibrosis and beta mutations are involved in beta-thalassemia disease (see Materials et Methods for references).
[±] Nonsense mutations are classified according to their gentamicin induced readthrough level.
