# Supplementary material for: Statistical Analysis of Readthrough Levels for Nonsense Mutations in Mammalian Cells Reveals a Major Determinant of Response to Gentamicin
Source: PLoS Genet. 2012 Mar 29;8(3):e1002608. doi: 10.1371/journal.pgen.1002608 (PMC3315467; doi:10.1371/journal.pgen.1002608)
Supplement: Table S2 — Descriptive statistics for the 3 parameters B, G and I before Box-Cox transformation. (PDF) [file pgen.1002608.s005.pdf]

Table S2: Descriptive statistics for the 3 parameters B, G and I (before Box-Cox transformation)

**Basal readthrough (B)**	
Mean	0.072%
Standard error of the mean	0.012%
Median	0.040%
Mode	0.040%
Standard deviation	0.098%
Sample variance	0.0001%
Kurstosis (Flattening coefficient)	11.11
Asymmetry coefficient	3.22
Plage	0.01
Minimum	0.01%
Maximum	0.53%
Sum	4.74%
Number	66
**Gentamicin-induced readthrough (G)**	
Mean	0.372%
Standard error of the mean	0.056%
Median	0.231%
Mode	0.200%
Standard deviation	0.458%
Sample variance	0.002%
Kurstosis (Flattening coefficient)	12.72
Asymmetry coefficient	3.23
Plage	0.03
Minimum	0.04%
Maximum	2.79%
Sum	24.53%
Number	66
**Increase factor (I)**	
Mean	6.0
Standard error of the mean	0.4
Median	5.5
Mode	3.0
Standard deviation	2.9
Sample variance	8.3
Kurstosis (Flattening coefficient)	1.6
Asymmetry coefficient	1.2
Plage	14.7
Minimum	1.6
Maximum	16.3
Sum	398.6
Number	66
