# Supplementary material for: Statistical Analysis of Readthrough Levels for Nonsense Mutations in Mammalian Cells Reveals a Major Determinant of Response to Gentamicin
Source: PLoS Genet. 2012 Mar 29;8(3):e1002608. doi: 10.1371/journal.pgen.1002608 (PMC3315467; doi:10.1371/journal.pgen.1002608)
Supplement: Table S3 — Shapiro Test after Box-Cox transformation (λ = −0.217). (PDF) [file pgen.1002608.s006.pdf]

Table S3 : Shapiro Test after Box-cox transformation (λ= -0.217)

Basal readthrough (B)	
W	0.988
p-value	0.794
alpha	0.05
Gentamicin readthrough(G)	
W	0.990
p-value	0.865
alpha	0.05
Increase Factor (I):	
W	0.990
p-value	0.859
alpha	0.05
