# Supplementary material for: Statistical Analysis of Readthrough Levels for Nonsense Mutations in Mammalian Cells Reveals a Major Determinant of Response to Gentamicin
Source: PLoS Genet. 2012 Mar 29;8(3):e1002608. doi: 10.1371/journal.pgen.1002608 (PMC3315467; doi:10.1371/journal.pgen.1002608)
Supplement: Table S4 — Bravais-Pearson statistical analysis of correlation between basal readthrough, induced readthrough and gentamicin response (Increase Factor) after Box-Cox transformation. (PDF) [file pgen.1002608.s007.pdf]

Table S4: Bravais-Pearson statistical analysis of correlation between basal readthrough, induced readthrough and gentamicin response (Increase Factor) after Box-Cox transformation

R

Variables	Basal	Gentamicin	Increase Factor
Basal	1	0.845	-0.296
Gentamicin		1	0.248
Increase Factor			1

p-values:

Variables	Basal	Gentamicin	Increase Factor
Basal		< 0.0001	0.016
Gentamicin			0.045
Increase Factor			
