# Supplementary material for: Statistical Analysis of Readthrough Levels for Nonsense Mutations in Mammalian Cells Reveals a Major Determinant of Response to Gentamicin
Source: PLoS Genet. 2012 Mar 29;8(3):e1002608. doi: 10.1371/journal.pgen.1002608 (PMC3315467; doi:10.1371/journal.pgen.1002608)
Supplement: Table S5 — Statistical analysis of the effect of the type of stop codon on B, G and I after Box-Cox transformation using a common lambda: −0.217 (this transformation leads to negative value for B and G). (PDF) [file pgen.1002608.s008.pdf]

Table S5: Statistical analysis of the effect of the type of stop codon on B, G and I after Box-Cox transformation using a common lambda: -0.217 (this transformation leads to negative value for B and G).

**Stop codon**	**Actual number**	**B**	**G**	**I**
**UGA** mean (variance)	27	-18.47 (13.82)	- 10.93 (4.15)	1.48 (0.09)
**UAG** mean (variance)	25	-20.73 (16.22)	- 13.43 (10.63)	1.33 (0.12)
**UAA** mean (variance)	14	-24.11 (31.96)	- 15.25 (10.11)	1.38 (0.10)
Bartlett test (p)		0.1755	0.0532	0.7404
ANOVA : F (2;63)=		**7.98, p=0.0008**	**12.00, p=0.000038**	1.47, p=0.238
LSD test (C.I. 93%)		**UGA>UAG>UAA**	**UGA>UAG>UAA**	/
