# Supplementary material for: Statistical Analysis of Readthrough Levels for Nonsense Mutations in Mammalian Cells Reveals a Major Determinant of Response to Gentamicin
Source: PLoS Genet. 2012 Mar 29;8(3):e1002608. doi: 10.1371/journal.pgen.1002608 (PMC3315467; doi:10.1371/journal.pgen.1002608)
Supplement: Table S6 — Statistical analysis of the effect of each nucleotide at each position on B, G and I after Box-Cox transformation using a common lambda: −0.217 (this transformation leads to negative value for B and G). (DOC) [file pgen.1002608.s009.doc]

**Table S6:** Statistical analysis of the effect of each nucleotide at each position on B, G and I after Box-Cox transformation using a common lambda: -0.217 (this transformation leads to negative value for B and G).

| **position**  **nucleotide** | **-6** | **-5** | **-4** | **-3** | **-2** | **-1** | **+4** | **+5** | **+6** | **+7** | **+8** | **+9** |
| --- | --- | --- | --- | --- | --- | --- | --- | --- | --- | --- | --- | --- |
| **A actual number** | 18 | 18 | 10 | 19 | 20 | 13 | 18 | 16 | 17 | 18 | 24 | 12 |
| **C actual number** | 19 | 18 | 13 | 19 | 11 | 17 | 15 | 18 | 15 | 19 | 10 | 20 |
| **G actual number** | 17 | 10 | 25 | 17 | 14 | 18 | 25 | 11 | 17 | 20 | 16 | 16 |
| **U actual number** | 12 | 20 | 18 | 11 | 21 | 18 | 8 | 21 | 17 | 9 | 16 | 18 |
| **Basal readthrough level (B)** | | | | | | | | | | | | |
| **A mean (variance)** | -19.29 (29.72) | -19.14 (19.60) | -19.95 (22.68) | -20.33 (13.72) | -20.84 (13.00) | -21.63 (30.66) | -21.23 (15.59) | -22.34 (35.37) | -19.94 (27.00) | -19.41 (12.95) | -20.02 (20.11) | -20.05 (16.01) |
| **C mean (variance)** | -19.34 (24.69) | -18.38 (14.29) | -20.76 (32.86) | -20.84 (21.05) | -20.51 (22.40) | -21.57 (15.37) | -17.66 (40.10) | -21.01 (19.81) | -19.14 (20.31) | -20.09 (14.43) | -22.48 (21.08) | -21.33 (34.60) |
| **G mean (variance)** | -21.28 (14.55) | -22.73 (4.48) | -20.84 (26.43) | -19.86 (23.28) | -20.20 (33.88) | -19.40 (17.94) | -21.32 (13.47) | -20.04 (10.86) | -20.41 (18.88) | -20.94 (35.89) | -19.18 (32.72) | -18.91 (15.84) |
| **U mean (variance)** | -23.19 (11.57) | -22.59 (31;12) | -20.24 (12.85) | -21.35 (44.31) | -20.45 (27.21) | -19.87 (27.96) | -21.84 (21.12) | -18.98 (18.69) | -20.18 (25.75) | - 22.75 (27.94) | -21.40 (15.38) | -21.39 (18.64) |
| **Bartlett test (p)** | 0.276 | 0.026 | 0.335 | 0.197 | 0.275 | 0.486 | 0.102 | 0.239 | 0.873 | 0.103 | 0.524 | 0.305 |
| **ANOVA : F(3;62)=** | 2.35, p=0.081 |  | 0.11,  p=0.952 | 0.25, p=0.859 | 0.05, p=0.985 | 0.96, p=0.416 | **2.57,**  **p=0.06** | 1.67,  p=0.18 | 0.15, p=0.931 | 1.10, p=0.355 | 1.28, p=0.288 | 1.06, p=0.374 |
| **LSD test (C.I. 95%)** | A>U - C>U |  | / | / | / | / | **C>A, G, U** | U>A | / | / | / | / |
| **Gentamicin readthrough level (G)** | | | | | | | | | | | | |
| **A mean (variance)** | 11.92 (9.40) | 12.14 (10.82) | 12.63 (12.57) | 12.68 (8.84) | 13.57 (8.95) | 14.52 (12.51) | 13.76 (8.10) | 12.82 (16.39) | 11.85 (6.74) | 12.65 (8,81) | 12.38 (8.03) | 12.44 (9.40) |
| **C mean (variance)** | 12.25 (17.88) | 11.58 (11.22) | 12.96 (13.19) | 12.74 (11.25) | 11.90 (9.57) | 13.16 (5.97) | 10.11 (13.50) | 12.87 (9.65) | 11.75 (8.17) | 12.66 (10.16) | 14.60 (12.99) | 13.93 (17.88) |
| **G mean (variance)** | 13.70 (4.81) | 15.02 (2.30) | 13.00 (10.39) | 13.45 (10.94) | 12.28 (8.92) | 13.04 (9.62) | 13.12 (6.08) | 13.21 (4.20) | 12.87 (9.71) | 12.55 (13.24) | 11.58 (14.80) | 12.13 (4.82) |
| **U mean (variance)** | 13.65 (7.61) | 13.34 (10.39) | 12.47 (9.32) | 12.04 (13.20) | 12.85 (13.82) | 10.94 (9.95) | 14.62 (7.92) | 12.47 (11.33) | 13.19 (17.69) | 13.88 (10.74) | 13.49 (6.06) | 12.35 (7.61) |
| **Bartlett test (p)** | 0.058 | 0.100 | 0.911 | 0.906 | 0.747 | 0.585 | 0.406 | 0.185 | 0.230 | 0.859 | 0.300 | 0.058 |
| **ANOVA : F(3;62)=** | 1.36, p=0.265 | 1.82, p=0.193 | 0.11,  p=0.954 | 0.43, p=0.731 | 0.77, p=0.517 | **3.73,**  **p=0.02** | **6.05, p=0.001** | 0.13, p=0.940 | 0.59, p=0.625 | 0.39, p=0.762 | 2.29, p=0.087 | 1.21, p=0.313 |
| **LSD test (C.I. 95%)** | / | A, C>G | / | / | / | **U>A, C, G** | **C>A, G, U** | / | / | / | G>C | / |
| **Increase Factor (I)** | | | | | | | | | | | | |
| **A mean (variance)** | 1.40 (0.07) | 1.34 (0.10) | 1.36 (0.11) | 1.41 (0.09) | 1.31 (0.11) | 1.21 (0.11) | 1.32 (0.08) | 1.61 (0.09) | 1.46 (0.11) | 1.30 (0.08) | 1.40 (0.12) | 1.42 (0.06) |
| **C mean (variance)** | 1.35 (0.12) | 1.36 (0.14) | 1.39 (0.09) | 1.45 (0.10) | 1.56 (0.07) | 1.47 (0.07) | 1.53 (0.08) | 1.45 (0.07) | 1.45 (0.09) | 1.37 (0.14) | 1.35 (0.05) | 1.30 (0.13) |
| **G mean (variance)** | 1.32 (0.13) | 1.30 (0.04) | 1.40 (0.10) | 1.18 (0.10) | 1.42 (0.16) | 1.21 (0.06) | 1.44 (0.09) | 1.27 (0.03) | 1.38 (0.10) | 1.47 (0.12) | 1.46 (0.07) | 1.30 (0.08) |
| **U mean (variance)** | 1.58 (0.09) | 1.53 (0.10) | 1.43 (0.14) | 1.62 (0.05) | 1.39 (0.07) | 1.66 (0.05) | 1.21 (0.20) | 1.26 (0.13) | 1.30 (0.13) | 1.49 (0.04) | 1.37 (0.17) | 1.58 (0.09) |
| **Bartlett test (p)** | 0.602 | 0.334 | 0.823 | 0.698 | 0.431 | 0.532 | 0.413 | 0.137 | 0.897 | 0.301 | 0.129 | 0.602 |
| **ANOVA : F(3;62)=** | 1.70, p=0.176 | 1.62, p=0.193 | 0.12,  p=0.949 | **5.17, p=0.003** | 1.48, p=0.229 | **11.16, p=0.000006** | 2.37, p=0.079 | **4.84, p=0.004** | 0.81, p=0.494 | 1.20, p=0.318 | 0.27, p=0.844 | **3.34, p=0.025** |
| **LSD test (C.I. 95%)** | U>G | / | / | **A, C, U>G** | C>A | **U>C>A, G** | C>U | **A>G, U** | / | / | / | **U>C,G** |
