# Supplementary material for: Statistical Analysis of Readthrough Levels for Nonsense Mutations in Mammalian Cells Reveals a Major Determinant of Response to Gentamicin
Source: PLoS Genet. 2012 Mar 29;8(3):e1002608. doi: 10.1371/journal.pgen.1002608 (PMC3315467; doi:10.1371/journal.pgen.1002608)
Supplement: Table S7 — Statistical analysis of the effect of nucleotide in −1 position on B, G and I (after Box-Cox transformation) restricting the pool of mutations to those without a C in +4. (DOC) [file pgen.1002608.s010.doc]

**Table S7:** Statistical analysis of the effect of nucleotide in -1 position on B, G and I (after Box-cox transformation) restricting the pool of mutations to those without a C in +4.

| **Nucleotide in -1 position** | **Actual number** | **Basal readthrough** | **Gentamicin readthrough** | **Increase Factor** |
| --- | --- | --- | --- | --- |
| **A mean (variance)** | 12 | -22.51 (22.35) | -15.06 (9.55) | 1.24 (0.11) |
| **C mean (variance)** | 11 | -20.82 (4.43) | -13.18 (2.32) | 1.38 (0.08) |
| **G mean (variance)** | 16 | -20.19 (12.87) | -13.59 (7.40) | 1.21 (0.07) |
| **U mean (variance)** | 12 | -22.30 (18.52) | -12.55 (5.58) | 1.64 (0.07) |
| **Bartlett test (p)** |  | 0.0956 | 0.1812 | 0.8215 |
| **ANOVA : F(3;47)=** |  | 1.18, p=0.329 | 2.12, p=0.110 | 6.16, p=0.001 |
| **LSD test (C.I. 95%)** |  | / | U>A | U>G, C, A |
