# Supplementary material for: Statistical Analysis of Readthrough Levels for Nonsense Mutations in Mammalian Cells Reveals a Major Determinant of Response to Gentamicin
Source: PLoS Genet. 2012 Mar 29;8(3):e1002608. doi: 10.1371/journal.pgen.1002608 (PMC3315467; doi:10.1371/journal.pgen.1002608)
Supplement: Table S8 — Statistical analysis of the effect of all nucleotides on B, G and I after Box-Cox transformation using a common lambda: −0.217. The significant differences between each nucleotide against all nucleotides at all positions are listed. In this list, inc is for Increase Factor A, G, C or U for the nucleotide and the − or + followed by a number for position. (DOC) [file pgen.1002608.s011.doc]

**Table S8:** Statistical analysis of the effect of all nucleotides on B. G and I after Box-Cox transformation using a common lambda: -0.217. The significant differences between each nucleotide against all nucleotides at all positions are listed. In this list, inc is for Increase Factor A, G, C or U for the nucleotide and the – or + followed by a number for position.

| **Basal** | | | | | |
| --- | --- | --- | --- | --- | --- |
| **Source of variation** | **Sum squares** | **DF** | **Mean square** | **F statistic** | **p** |
| **Groups** | 1024 | 47 | 22 | 0.98 | 0.51 |
| **Residual** | 16486 | 744 | 22 |  |  |
| **Total** | 17510 | 791 |  |  |  |
|  |  |  |  |  |  |
| **Gentamicin** | | | | | |
| **Source of variation** | **Sum squares** | **DF** | **Mean square** | **F statistic** | **p** |
| **Groups** | 578 | 47 | 12 | 1.20 | 0.17 |
| **Residual** | 7614 | 744 | 10 |  |  |
| **Total** | 8192 | 791 |  |  |  |
|  |  |  |  |  |  |
| **Increase Factor** | | | | | |
| **Source of variation** | **Sum squares** | **DF** | **Mean square** | **F statistic** | **p** |
| **Groups** | 9 | 47 | 0.19 | 1.94 | 0.0002 |
| **Residual** | 74 | 744 | 0.10 |  |  |
| **Total** | 83 | 791 |  |  |  |

| **LSD Contrast** | **Difference** | **95% CI** | |  |
| --- | --- | --- | --- | --- |
| incA-6 v incG-3 | 0.22 | 0.01 | 0.43 | (significant) |
| incA-6 v incU-1 | -0.25 | -0.46 | -0.05 | (significant) |
| incC-6 v incU-3 | -0.26 | -0.50 | -0.03 | (significant) |
| incC-6 v incU-1 | -0.30 | -0.51 | -0.10 | (significant) |
| incC-6 v incA+5 | -0.25 | -0.46 | -0.04 | (significant) |
| incC-6 v incU+9 | -0.23 | -0.43 | -0.03 | (significant) |
| incG-6 v incU-6 | -0.26 | -0.49 | -0.02 | (significant) |
| incG-6 v incU-5 | -0.21 | -0.41 | -0.01 | (significant) |
| incG-6 v incU-3 | -0.30 | -0.54 | -0.06 | (significant) |
| incG-6 v incC-2 | -0.24 | -0.48 | 0.00 | (significant) |
| incG-6 v incU-1 | -0.34 | -0.55 | -0.13 | (significant) |
| incG-6 v incA+5 | -0.29 | -0.50 | -0.07 | (significant) |
| incG-6 v incU+9 | -0.27 | -0.48 | -0.06 | (significant) |
| incU-6 v incA-5 | 0.24 | 0.01 | 0.47 | (significant) |
| incU-6 v incG-5 | 0.27 | 0.01 | 0.54 | (significant) |
| incU-6 v incG-3 | 0.39 | 0.16 | 0.63 | (significant) |
| incU-6 v incA-2 | 0.27 | 0.04 | 0.49 | (significant) |
| incU-6 v incA-1 | 0.36 | 0.12 | 0.61 | (significant) |
| incU-6 v incG-1 | 0.37 | 0.14 | 0.60 | (significant) |
| incU-6 v incA+4 | 0.26 | 0.03 | 0.49 | (significant) |
| incU-6 v incU+4 | 0.36 | 0.08 | 0.65 | (significant) |
| incU-6 v incG+5 | 0.31 | 0.05 | 0.56 | (significant) |
| incU-6 v incU+5 | 0.31 | 0.09 | 0.54 | (significant) |
| incU-6 v incU+6 | 0.27 | 0.04 | 0.50 | (significant) |
| incU-6 v incA+7 | 0.28 | 0.05 | 0.51 | (significant) |
| incU-6 v incC+9 | 0.27 | 0.05 | 0.50 | (significant) |
| incU-6 v incG+9 | 0.27 | 0.04 | 0.51 | (significant) |
| incA-5 v incU-3 | -0.28 | -0.52 | -0.04 | (significant) |
| incA-5 v incU-1 | -0.32 | -0.52 | -0.11 | (significant) |
| incA-5 v incA+5 | -0.27 | -0.48 | -0.05 | (significant) |
| incA-5 v incU+9 | -0.24 | -0.45 | -0.04 | (significant) |
| incC-5 v incU-3 | -0.25 | -0.49 | -0.01 | (significant) |
| incC-5 v incU-1 | -0.29 | -0.50 | -0.08 | (significant) |
| incC-5 v incA+5 | -0.24 | -0.45 | -0.03 | (significant) |
| incC-5 v incU+9 | -0.22 | -0.42 | -0.01 | (significant) |
| incG-5 v incU-3 | -0.32 | -0.59 | -0.05 | (significant) |
| incG-5 v incU-1 | -0.36 | -0.60 | -0.11 | (significant) |
| incG-5 v incA+5 | -0.30 | -0.55 | -0.06 | (significant) |
| incG-5 v incU+9 | -0.28 | -0.53 | -0.04 | (significant) |
| incU-5 v incG-3 | 0.34 | 0.14 | 0.55 | (significant) |
| incU-5 v incA-2 | 0.22 | 0.02 | 0.41 | (significant) |
| incU-5 v incA-1 | 0.32 | 0.10 | 0.54 | (significant) |
| incU-5 v incG-1 | 0.32 | 0.12 | 0.52 | (significant) |
| incU-5 v incA+4 | 0.21 | 0.01 | 0.41 | (significant) |
| incU-5 v incU+4 | 0.32 | 0.06 | 0.57 | (significant) |
| incU-5 v incG+5 | 0.26 | 0.03 | 0.49 | (significant) |
| incU-5 v incU+5 | 0.26 | 0.07 | 0.46 | (significant) |
| incU-5 v incU+6 | 0.22 | 0.02 | 0.43 | (significant) |
| incU-5 v incA+7 | 0.23 | 0.03 | 0.43 | (significant) |
| incU-5 v incC+9 | 0.23 | 0.03 | 0.42 | (significant) |
| incU-5 v incG+9 | 0.23 | 0.02 | 0.43 | (significant) |
| incA-4 v incU-1 | -0.30 | -0.54 | -0.05 | (significant) |
| incC-4 v incU-1 | -0.27 | -0.50 | -0.05 | (significant) |
| incG-4 v incG-3 | 0.21 | 0.02 | 0.40 | (significant) |
| incG-4 v incU-3 | -0.22 | -0.45 | 0.00 | (significant) |
| incG-4 v incU-1 | -0.26 | -0.46 | -0.07 | (significant) |
| incG-4 v incA+5 | -0.21 | -0.41 | -0.01 | (significant) |
| incU-4 v incG-3 | 0.25 | 0.04 | 0.46 | (significant) |
| incU-4 v incG-1 | 0.23 | 0.02 | 0.43 | (significant) |
| incU-4 v incU-1 | -0.22 | -0.43 | -0.02 | (significant) |
| incA-3 v incG-3 | 0.23 | 0.02 | 0.43 | (significant) |
| incA-3 v incG-1 | 0.20 | 0.00 | 0.41 | (significant) |
| incA-3 v incU-1 | -0.25 | -0.45 | -0.04 | (significant) |
| incC-3 v incG-3 | 0.27 | 0.06 | 0.48 | (significant) |
| incC-3 v incA-1 | 0.24 | 0.02 | 0.46 | (significant) |
| incC-3 v incG-1 | 0.25 | 0.04 | 0.45 | (significant) |
| incC-3 v incU-1 | -0.21 | -0.41 | 0.00 | (significant) |
| incG-3 v incU-3 | -0.44 | -0.67 | -0.20 | (significant) |
| incG-3 v incC-2 | -0.38 | -0.62 | -0.14 | (significant) |
| incG-3 v incG-2 | -0.24 | -0.46 | -0.02 | (significant) |
| incG-3 v incU-2 | -0.20 | -0.40 | 0.00 | (significant) |
| incG-3 v incC-1 | -0.29 | -0.50 | -0.08 | (significant) |
| incG-3 v incU-1 | -0.48 | -0.68 | -0.27 | (significant) |
| incG-3 v incC+4 | -0.35 | -0.57 | -0.13 | (significant) |
| incG-3 v incG+4 | -0.26 | -0.45 | -0.06 | (significant) |
| incG-3 v incA+5 | -0.42 | -0.64 | -0.21 | (significant) |
| incG-3 v incC+5 | -0.27 | -0.48 | -0.06 | (significant) |
| incG-3 v incA+6 | -0.28 | -0.49 | -0.07 | (significant) |
| incG-3 v incC+6 | -0.27 | -0.49 | -0.05 | (significant) |
| incG-3 v incG+7 | -0.29 | -0.49 | -0.09 | (significant) |
| incG-3 v incU+7 | -0.31 | -0.56 | -0.05 | (significant) |
| incG-3 v incA+8 | -0.21 | -0.41 | -0.02 | (significant) |
| incG-3 v incG+8 | -0.27 | -0.49 | -0.06 | (significant) |
| incG-3 v incU+9 | -0.40 | -0.61 | -0.19 | (significant) |
| incU-3 v incA-2 | 0.31 | 0.08 | 0.54 | (significant) |
| incU-3 v incU-2 | 0.23 | 0.00 | 0.46 | (significant) |
| incU-3 v incA-1 | 0.41 | 0.15 | 0.66 | (significant) |
| incU-3 v incG-1 | 0.41 | 0.18 | 0.65 | (significant) |
| incU-3 v incA+4 | 0.30 | 0.07 | 0.54 | (significant) |
| incU-3 v incU+4 | 0.41 | 0.12 | 0.69 | (significant) |
| incU-3 v incG+5 | 0.35 | 0.09 | 0.61 | (significant) |
| incU-3 v incU+5 | 0.36 | 0.13 | 0.59 | (significant) |
| incU-3 v incU+6 | 0.31 | 0.08 | 0.55 | (significant) |
| incU-3 v incA+7 | 0.32 | 0.08 | 0.56 | (significant) |
| incU-3 v incC+7 | 0.25 | 0.01 | 0.48 | (significant) |
| incU-3 v incU+8 | 0.25 | 0.00 | 0.49 | (significant) |
| incU-3 v incC+9 | 0.32 | 0.09 | 0.55 | (significant) |
| incU-3 v incG+9 | 0.32 | 0.08 | 0.56 | (significant) |
| incA-2 v incC-2 | -0.25 | -0.48 | -0.02 | (significant) |
| incA-2 v incU-1 | -0.35 | -0.55 | -0.15 | (significant) |
| incA-2 v incC+4 | -0.22 | -0.43 | -0.01 | (significant) |
| incA-2 v incA+5 | -0.30 | -0.51 | -0.09 | (significant) |
| incA-2 v incU+9 | -0.28 | -0.48 | -0.07 | (significant) |
| incC-2 v incA-1 | 0.35 | 0.10 | 0.60 | (significant) |
| incC-2 v incG-1 | 0.35 | 0.12 | 0.59 | (significant) |
| incC-2 v incA+4 | 0.25 | 0.01 | 0.48 | (significant) |
| incC-2 v incU+4 | 0.35 | 0.06 | 0.64 | (significant) |
| incC-2 v incG+5 | 0.29 | 0.03 | 0.55 | (significant) |
| incC-2 v incU+5 | 0.30 | 0.07 | 0.53 | (significant) |
| incC-2 v incU+6 | 0.26 | 0.02 | 0.50 | (significant) |
| incC-2 v incA+7 | 0.26 | 0.02 | 0.50 | (significant) |
| incC-2 v incC+9 | 0.26 | 0.03 | 0.49 | (significant) |
| incC-2 v incG+9 | 0.26 | 0.02 | 0.50 | (significant) |
| incG-2 v incU-1 | -0.24 | -0.46 | -0.02 | (significant) |
| incU-2 v incU-1 | -0.27 | -0.47 | -0.07 | (significant) |
| incU-2 v incA+5 | -0.22 | -0.43 | -0.02 | (significant) |
| incA-1 v incC-1 | -0.26 | -0.49 | -0.03 | (significant) |
| incA-1 v incU-1 | -0.45 | -0.67 | -0.22 | (significant) |
| incA-1 v incC+4 | -0.32 | -0.55 | -0.08 | (significant) |
| incA-1 v incG+4 | -0.23 | -0.44 | -0.02 | (significant) |
| incA-1 v incA+5 | -0.39 | -0.63 | -0.16 | (significant) |
| incA-1 v incC+5 | -0.24 | -0.46 | -0.01 | (significant) |
| incA-1 v incA+6 | -0.25 | -0.48 | -0.02 | (significant) |
| incA-1 v incC+6 | -0.24 | -0.47 | 0.00 | (significant) |
| incA-1 v incG+7 | -0.26 | -0.48 | -0.04 | (significant) |
| incA-1 v incU+7 | -0.28 | -0.55 | -0.01 | (significant) |
| incA-1 v incG+8 | -0.25 | -0.48 | -0.02 | (significant) |
| incA-1 v incU+9 | -0.37 | -0.60 | -0.15 | (significant) |
| incC-1 v incG-1 | 0.26 | 0.05 | 0.47 | (significant) |
| incC-1 v incU+5 | 0.21 | 0.01 | 0.41 | (significant) |
| incG-1 v incU-1 | -0.45 | -0.66 | -0.25 | (significant) |
| incG-1 v incC+4 | -0.32 | -0.54 | -0.11 | (significant) |
| incG-1 v incG+4 | -0.23 | -0.42 | -0.04 | (significant) |
| incG-1 v incA+5 | -0.40 | -0.61 | -0.19 | (significant) |
| incG-1 v incC+5 | -0.24 | -0.45 | -0.04 | (significant) |
| incG-1 v incA+6 | -0.25 | -0.46 | -0.04 | (significant) |
| incG-1 v incC+6 | -0.24 | -0.46 | -0.03 | (significant) |
| incG-1 v incG+7 | -0.27 | -0.47 | -0.07 | (significant) |
| incG-1 v incU+7 | -0.28 | -0.54 | -0.03 | (significant) |
| incG-1 v incG+8 | -0.25 | -0.46 | -0.04 | (significant) |
| incG-1 v incU+9 | -0.38 | -0.58 | -0.17 | (significant) |
| incU-1 v incA+4 | 0.34 | 0.14 | 0.55 | (significant) |
| incU-1 v incG+4 | 0.22 | 0.03 | 0.41 | (significant) |
| incU-1 v incU+4 | 0.45 | 0.18 | 0.71 | (significant) |
| incU-1 v incC+5 | 0.21 | 0.00 | 0.41 | (significant) |
| incU-1 v incG+5 | 0.39 | 0.15 | 0.63 | (significant) |
| incU-1 v incU+5 | 0.40 | 0.20 | 0.59 | (significant) |
| incU-1 v incG+6 | 0.27 | 0.06 | 0.48 | (significant) |
| incU-1 v incU+6 | 0.35 | 0.15 | 0.56 | (significant) |
| incU-1 v incA+7 | 0.36 | 0.15 | 0.57 | (significant) |
| incU-1 v incC+7 | 0.29 | 0.08 | 0.49 | (significant) |
| incU-1 v incA+8 | 0.26 | 0.07 | 0.45 | (significant) |
| incU-1 v incC+8 | 0.31 | 0.06 | 0.55 | (significant) |
| incU-1 v incU+8 | 0.29 | 0.07 | 0.50 | (significant) |
| incU-1 v incA+9 | 0.24 | 0.01 | 0.48 | (significant) |
| incU-1 v incC+9 | 0.36 | 0.16 | 0.56 | (significant) |
| incU-1 v incG+9 | 0.36 | 0.14 | 0.57 | (significant) |
| incA+4 v incA+5 | -0.29 | -0.50 | -0.08 | (significant) |
| incA+4 v incU+9 | -0.27 | -0.48 | -0.06 | (significant) |
| incC+4 v incU+4 | 0.32 | 0.05 | 0.59 | (significant) |
| incC+4 v incG+5 | 0.26 | 0.02 | 0.51 | (significant) |
| incC+4 v incU+5 | 0.27 | 0.06 | 0.48 | (significant) |
| incC+4 v incU+6 | 0.23 | 0.01 | 0.45 | (significant) |
| incC+4 v incA+7 | 0.23 | 0.02 | 0.45 | (significant) |
| incC+4 v incC+9 | 0.23 | 0.02 | 0.44 | (significant) |
| incC+4 v incG+9 | 0.23 | 0.01 | 0.45 | (significant) |
| incU+4 v incA+5 | -0.39 | -0.66 | -0.13 | (significant) |
| incU+4 v incG+7 | -0.26 | -0.52 | 0.00 | (significant) |
| incU+4 v incU+9 | -0.37 | -0.63 | -0.11 | (significant) |
| incA+5 v incG+5 | 0.34 | 0.09 | 0.58 | (significant) |
| incA+5 v incU+5 | 0.34 | 0.14 | 0.55 | (significant) |
| incA+5 v incG+6 | 0.22 | 0.00 | 0.44 | (significant) |
| incA+5 v incU+6 | 0.30 | 0.09 | 0.52 | (significant) |
| incA+5 v incA+7 | 0.31 | 0.09 | 0.52 | (significant) |
| incA+5 v incC+7 | 0.23 | 0.03 | 0.44 | (significant) |
| incA+5 v incA+8 | 0.21 | 0.01 | 0.41 | (significant) |
| incA+5 v incC+8 | 0.26 | 0.01 | 0.51 | (significant) |
| incA+5 v incU+8 | 0.23 | 0.02 | 0.45 | (significant) |
| incA+5 v incC+9 | 0.31 | 0.10 | 0.51 | (significant) |
| incA+5 v incG+9 | 0.31 | 0.09 | 0.52 | (significant) |
| incG+5 v incU+9 | -0.31 | -0.55 | -0.08 | (significant) |
| incU+5 v incG+7 | -0.21 | -0.40 | -0.02 | (significant) |
| incU+5 v incU+9 | -0.32 | -0.52 | -0.12 | (significant) |
| incU+6 v incU+9 | -0.28 | -0.49 | -0.07 | (significant) |
| incA+7 v incU+9 | -0.28 | -0.49 | -0.08 | (significant) |
| incC+7 v incU+9 | -0.21 | -0.42 | -0.01 | (significant) |
| incC+9 v incU+9 | -0.28 | -0.48 | -0.08 | (significant) |
| incG+9 v incU+9 | -0.28 | -0.49 | -0.07 | (significant) |
