# Supplementary material for: Statistical Analysis of Readthrough Levels for Nonsense Mutations in Mammalian Cells Reveals a Major Determinant of Response to Gentamicin
Source: PLoS Genet. 2012 Mar 29;8(3):e1002608. doi: 10.1371/journal.pgen.1002608 (PMC3315467; doi:10.1371/journal.pgen.1002608)
Supplement: Table S9 — Statistical data (Standard deviation and standard error of the mean) of studied nonsense mutations (Figure 7 and Figure 8). (DOC) [file pgen.1002608.s012.doc]

**Table S9**: Statistical data of studied nonsense mutations (Figure 7 and 8)

| **DMD 673** | | **Mean** | **Median** | **SD^¥^** | **SEM^£^** |
| --- | --- | --- | --- | --- | --- |
| **A** | 0 | 0.49% | 0.45% | 0.26% | 0.11% |
|  | gentamicin | 0.91% | 0.84% | 0.36% | 0.15% |
| **C** | 0 | 0.29% | 0.27% | 0.10% | 0.05% |
|  | gentamicin | 0.61% | 0.58% | 0.16% | 0.07% |
| **G** | 0 | 0.43% | 0.45% | 0.18% | 0.09% |
|  | gentamicin | 0.93% | 0.80% | 0.23% | 0.12% |
| **U** | 0 | 0.11% | 0.11% | 0.04 % | 0.02% |
|  | gentamicin | 0.57% | 0.55% | 0.27% | 0.13% |

| **DMD 319** |  | **Mean** | **Median** | **SD** | **SEM** |
| --- | --- | --- | --- | --- | --- |
| **A** | 0 | 0.16% | 0.13% | 0.08% | 0.03% |
|  | gentamicin | 0.38% | 0.39% | 0.11% | 0.05% |
| **C** | 0 | 0.08% | 0.08% | 0.01% | 0.004% |
|  | gentamicin | 0.36% | 0.33% | 0.06% | 0,03% |
| **G** | 0 | 0.11% | 0.10% | 0.04% | 0.02% |
|  | gentamicin | 0.35% | 0.32% | 0.09% | 0.04% |
| **U** | 0 | 0.06% | 0.06% | 0.02% | 0.01% |
|  | gentamicin | 0.53% | 0.55% | 0.12% | 0.05% |

| **CF 122** | | **Mean** | **Median** | **SD** | **SEM** |
| --- | --- | --- | --- | --- | --- |
| **G** | 0 | 0.52% | 0.51% | 0.13% | 0.06% |
|  | gentamicin | 1.71% | 1.70% | 0.10% | 0.04% |
| **U** | 0 | 0.12% | 0.10% | 0.05% | 0.02% |
|  | gentamicin | 0.70% | 0.75% | 0.29% | 0.10% |

| **DMD 931** | | **Mean** | **Median** | **SD** | **SEM** |
| --- | --- | --- | --- | --- | --- |
| **A** | 0 | 0.25% | 0.37% | 0.08% | 0.04% |
|  | gentamicin | 0.94% | 0.90% | 0.25% | 0.08% |
| **U** | 0 | 0.11% | 0.11% | 0.03% | 0.01% |
|  | gentamicin | 0.99% | 0.82% | 0.34% | 0.12% |

| **p53 146** | | **Mean** | **Median** | **SD** | **SEM** |
| --- | --- | --- | --- | --- | --- |
| **G** | 0 | 0.06% | 0.05% | 0.01% | 0.005% |
|  | gentamicin | 0.24% | 0.26% | 0.04% | 0.02% |
| **U** | 0 | 0.02% | 0.01% | 0.004% | 0.002% |
|  | gentamicin | 0.19% | 0.21% | 0.05% | 0.02% |

| **beta 17** | | **Mean** | **Median** | **SD** | **SEM** |
| --- | --- | --- | --- | --- | --- |
| **C** | 0 | 0.02% | 0.02% | 0.01% | 0.003% |
|  | gentamicin | 0.7% | 0.07% | 0.02% | 0.008% |
| **U** | 0 | 0.02% | 0.02% | 0.01% | 0.002% |
|  | gentamicin | 0.17% | 0.16% | 0.07% | 0.03% |

| **APC 1114** | | **Mean** | **Median** | **SD** | **SEM** |
| --- | --- | --- | --- | --- | --- |
| **C** | 0 | 0.05% | 0.05% | 0.01% | 0.006% |
|  | gentamicin | 0.21% | 0.21% | 0.04% | 0.02% |
| **U** | 0 | 0.07% | 0.06% | 0.02% | 0.01% |
|  | gentamicin | 0.73% | 0.61% | 0.06% | 0.03% |

| **APC 1131** | | **Mean** | **Median** | **SD** | **SEM** |
| --- | --- | --- | --- | --- | --- |
|  | 0 | 0.01% | 0.01% | 0.002% | 0.001% |
| **C** | gentamicin | 0.05% | 0.05% | 0.03% | 0.01% |
|  | 0 | 0.01% | 0.01% | 0.002% | 0.001% |
| **U** | gentamicin | 0.18% | 0.19% | 0.03% | 0.01% |

^¥^ SD: Standard deviation

^£^ SEM: Standard error of the mean
